# Forecasting municipal solid plastic waste generation and management policy using system dynamics: a case study of Khulna City in Bangladesh

**DOI:** 10.1007/s10661-024-12684-1

**Published:** 2024-05-14

**Authors:** Islam M. Rafizul, Eckhard Kraft, Thomas Haupt, S. M. Rafew

**Affiliations:** 1 Department of Civil Engineering, Khulna University of Engineering and Technology, Khulna-9203, Bangladesh; 2 Biotechnology in Resources Management, Faculty of Civil Engineering, Bauhaus-Universität Weimar, Coudraystr. 7, 99423 Weimar, Germany

**Keywords:** Municipal solid plastic waste generation, Management, System dynamics, Policy, Khulna City

## Abstract

A comprehensive analysis of municipal solid plastic waste (MSPW) management while emphasizing plastic pollution severity in coastal cities around the world is mandatory to alleviate the augmenting plastic waste footprint in nature. Thus, decision-makers’ persuasion for numerous management solutions of MSPW flow-control can be met through meditative systematic strategies at the regional level. To forecast solutions focused on systematic policies, an agent-based system dynamics (ASD) model has been developed and simulated from 2023 to 2040 while considering significant knit parameters for MSPW management of Khulna City in Bangladesh. Baseline simulation results show that per-capita plastic waste generation will increase to 11.6 kg by 2040 from 8.92 kg in 2023. Eventually, the landfilled quantity of plastic waste has accumulated to 70,000 tons within 18 years. Moreover, the riverine discharge has increased to 834 tons in 2040 from a baseline quantity of 512 tons in 2023. So the plastic waste footprint index (PWFI) value rises to 24 by 2040. Furthermore, the absence of technological initiatives is responsible for the logarithmic rise of non-recyclable plastic waste to 1.35*1000=1350 tons. Finally, two consecutive policy scenarios with baseline factors such as controlled riverine discharge, increased collection and separation of plastic waste, expansion of recycle business, and locally achievable plastic conversion technologies have been simulated. Therefore, policy 2, with 69% conversion, 80% source separation, and 50% riverine discharge reduction of MSPW, has been found adequate from a sustainability perspective with the lowest PWFI ranges of 3.97 to 1.07 alongside a per-capita MSPW generation of 7.63 to 10 kg from 2023 till 2040.

**Graphical abstract:**

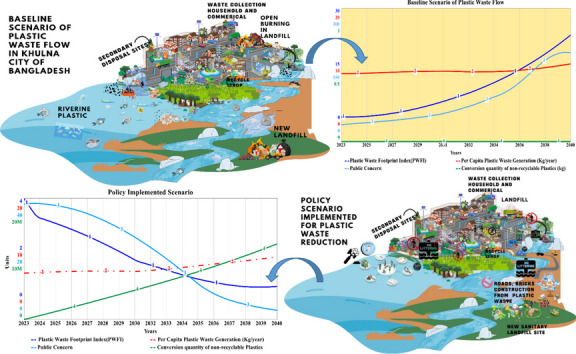

**Supplementary Information:**

The online version contains supplementary material available at 10.1007/s10661-024-12684-1.

## Introduction

The word plastic has originated from “pliable,” which can be defined as “easily shaped” (Evode et al., 2021). Since the beginning of the twentieth century, plastic has become an inescapable material in our everyday lives due to its affordability, malleability, lightweight, and non-corrosive properties. However, the omnipresence of plastics over the decades has been posing disposal challenges while accumulating in landfills and the natural environment (Law et al., 2020). A report from the United Nations Environmental Programme (UNEP) has stated that global plastic production has risen exponentially to 438 million tons until 2017(United Nations Environment Programme 2023). Moreover, the report has been designed to aid the decision makers on the necessary policy measures while articulating the BAU (Business as usual) scenario and systems change scenario by 2040. Therefore, this particular study on plastic waste generation and flow management in Khulna City is the first of its kind from a regional perspective to provide a quantitative perspective for the decision makers of Bangladesh to turn off the plastic flow tap and impose effective policy measures.

Klemeš et al. (2021) first proposed the concept of plastic waste footprints while focusing on the plastic products’ life cycle for facilitating decisions on plastic reduction. Recently, a large amount of valuable work has been carried out regarding the flow characteristics of plastic waste and its impact on the socioeconomic system at a global, national, and regional scale (Ciacci et al., 2017; Jambeck et al., 2015a, 2015b). At the national and regional scale, Eygen et al. (2017) and Kawecki et al. (2018) quantified the flow of major plastic waste in Austria and the EU respectively and recommended recycling as well as partial energy conversion. Boucher et al. (2019) have stated, “A national plastic footprint consists of the sum of domestic plastic pollution that serves domestic consumption and foreign plastic pollution that serves consumption in that particular country.” He further added that plastic footprint–related studies are divided mainly between emission-based and resource-based footprint assessments or between life cycle (LCA)–based and environmentally-extended input–output analysis (EEIOA) approaches. A recent study by Guillotreau et al. (2023) has applied the EEIOA approach to measure regional plastic waste footprint in small island developing states. The study has found the method to be more appropriate for assisting policy-makers to emphasize on particular sectors for reducing the municipal plastic waste footprint. In spite of such a heyday concept, that study failed to address the complexity of such a system and the estimation procedures. This study on Khulna City of Bangladesh with an agent-based system dynamics model can revolutionize not only multi-dimensional complex estimation procedure but also can simulate definitive policy measures to restrict the mass flow of macro plastic circulation in the environment and thus reduce plastic waste footprint.

A common phenomenon around the world is that we are implementing policies and legislations that just do not work or that, even more insidiously, the problem is solved in the short run and then it comes back repeatedly. That not only consumes valuable time but also does serious financial harm to hard-earned taxpayers’ money, which is eventually thwarting for sustainability. Thus, system dynamics gives us the means to anticipate some of those reactions and design higher-level impacts that can lead to sustainable success in the long run. In this regard, system dynamics simulation for forecasting plastic waste generation and for policy testing can play an insignificant role (Sterman et al., 2015). The simulation results will help policymakers understand the baseline and future policy scenarios from quantitative perspectives. According to a recent study by Liu et al. (2023), the quantity of plastic as well as disposable plastic products has been rising continuously due to the unavailability of alternative products, thus giving rise to the global carbon footprint of plastics by means of greenhouse gas emission. This global phenomenon requires further quantum cultivation of time-demanding management models from the regional level to control plastic pollution. This is where system dynamics can be applied due to its multi-variate factorial features, which rather than discrete components, considers as well as simulate the system as a whole (Sterman, 2000).

Thus, this study is aimed at developing an agent-based system dynamics (ASD) model to forecast as well as simulate Khulna City’s futuristic MSPW generation and management policy initiatives respectively for a time period from 2023 to 2040. It has been done through simulating the best possible policy scenario to manage and alleviate plastic waste flow in the city area. Finally, this ASD model and methodology has been developed while keeping in mind the implementation potential for identical spatiotemporal locations, specifically developing countries’ municipalities around the world.

## Literature review

System dynamics modelling is a modelling approach pioneered by Jay W. Forrester in the 1950s. Since then, the application has widened to numerous engineering fields such as MSW management, plastic waste management and waste to energy (Al-Shihabi et al., 2023; Bala et al., 2017; Rafew & Rafizul, 2021, 2023), ecology, forestry and environment (Sharifabadi, 2017), urban water resource policy management (Quezada et al., 2016), urban transportation and air pollution industry (Vafa-Arani et al., 2014), transportation, earthquake engineering (Pitilakis et al., 2008), earth’s climate, healthcare system, food industry, military, human resource management (Currie et al., 2018), cosmology (Bahamonde et al., 2018), and demolished structure (Mak et al., 2019). The agent-based system dynamics modelling concept has been in practice since the start of this millennium, as a handful number of pioneering studies by Scholl (2001). Schieritz (2002) established the earlier foundation of such work. A recent study by Latuszynska and Fate (2022) has stated that the complexity regarding the macro- and micro-scale parameters or factors is distinct in nature. To quantify such parameters within a single interconnected system, agent-based system dynamics simulation can provide deviated results. As a result, it is evident to assess further appropriate yet conjugal methods for incorporating macro- and microplastics due to the multi-dimensional continuous nature of a time-series simulation (Zulkepli & Eldabi, 2015). A recent study by Sethuraman et al. (2023) has used agent-based system dynamics modelling to link the emergent sequences of system-level processes for combating plastic waste pollution and its emission to oceans through a circular economic approach in USA.

Eventually, to impose regional policy, it is necessary to analyze the adequacy of the existing policies of that region. Thus, while analyzing the national action plan of Bangladesh, it has been observed that a 50% plastic recycling target by 2025 is already there. Additionally, a 90% phasing out the initiative of single-use plastic by 2026 and reducing total plastic waste to 30% by 2030 has already been mandated (Bangladesh Planning Commission, 2020). To achieve these goals, systematic management of plastic waste must come from municipalities, small towns, and communities, which will eventually be consolidated at the national level.

In terms of mismanaged plastic waste discharge to the marine environment, the Philippines tops the list with 7.71%, with Malaysia and Sri Lanka at second and third positions, respectively, with 4.38% and 3.42% (Meijer et al., 2021). For Asia, the per capita plastic waste emitted into the ocean is the highest in the Philippines and Malaysia, which is 3.3 kg and 2.29 kg per person per year, respectively (Jambeck et al., 2015a, 2015b). Bangladesh has been listed as one of the top 20 countries that generates plastics with less or no management tires (Law et al., 2020). Plastic pollution in Bangladesh has been increasing with the escalation of urbanization trends, and according to a study by Yoshijima et al. (2021), the per capita plastic waste generation in the country is 9.0 kg per person per year. A study by Ahsan et al. (2015) for Khulna City has found that institutions (Govt. and non-govt. office, education centers, and training centers) are the biggest contributors to plastic waste, with 14% of the total wet weight, whereas commercial and residential sources account for 9.1% and 2.0% respectively. The physical composition analysis has finally indicated a 3.1% plastic waste of the total generated municipal solid waste (MSW). Recent studies by Jodder et al. (2022) and Noman et al. (2023) have assessed the household solid waste generation and management scenario of eleven wards of Khulna City, where they found an avg. of 3.28% for three municipal wards and 4.5% of plastic waste from the total household waste weight (wet) percentage of 9 municipal wards respectively. Both the above-stated studies have also found significant correlations between waste generation and the income level of the inhabitants. It has been estimated that plastic discharge to the Bay of Bengal from rivers of Bangladesh and India through Sundarbans is 4 million tons/year and the project accumulated discharge by 2060 is expected to be 55 million tons (Adyel & Macreadie, 2021; Kumar et al., 2022; Lebreton & Andrady, 2019). Furthermore, numerous studies show that the Ganges is second in the top 20 plastic-polluted rivers, with a yearly average plastic discharge of 2.08 × 10^4^ m^3^s^−1^ (Chowdhury et al., 2020; Lebreton et al., 2017). Additionally, the articles also state that total marine plastic debris in coastal areas from Bangladesh has been 0.12–0.31 million metric tons per year, where pet bottles, LDPE, and HDPE are the most common forms of litter. According to a study by Meijer et al. (2021), 36 rivers of Bangladesh are among the 1656 rivers globally that contribute to global 80% of riverine plastic emissions. Being two adjacent rivers in the coastal belt, Rupsha and Bhairab are listed among the above-stated count. Macro plastic is the dominant component of river plastic pollution, and understanding these characteristics is pivotal for guiding the formulation and execution of further policies (Hurley et al., 2023; Rognerud et al., 2022). These statistics show the ever-deteriorating yet invisible nature of riverine plastic pollution in this region.

Kinds of literature regarding system dynamics for plastic waste generation and management is relatively limited. A recent study by Al-Shihabi et al. (2023) emphasized the policy measures to increase the net profit of technological conversion of discarded plastics while controlling the flow of plastic waste in Dubai, UAE, using system dynamics. Some aspects and features of such heyday studies have been incorporated in this particular study for plastic waste management of Khulna City while using a real-time dataset. A progressive study by Wang et al. (2022) integrated GIS, system dynamics, and AHP for determining standard operational procedures to select suitable waste treatment stations. Another study by Wongprapinkul and Vassanadumrongdee (2022) has used a systems thinking approach for single-use plastics in Thailand’s food delivery business. However, this study is the first of its kind in spatio-temporal aspect of not only for Khulna but also for Bangladesh to concentrate on the MSPW flow and policy measures at the same time through agent-based system dynamics technique.

## Materials and methodology

### Study area

**Fig. 1:**
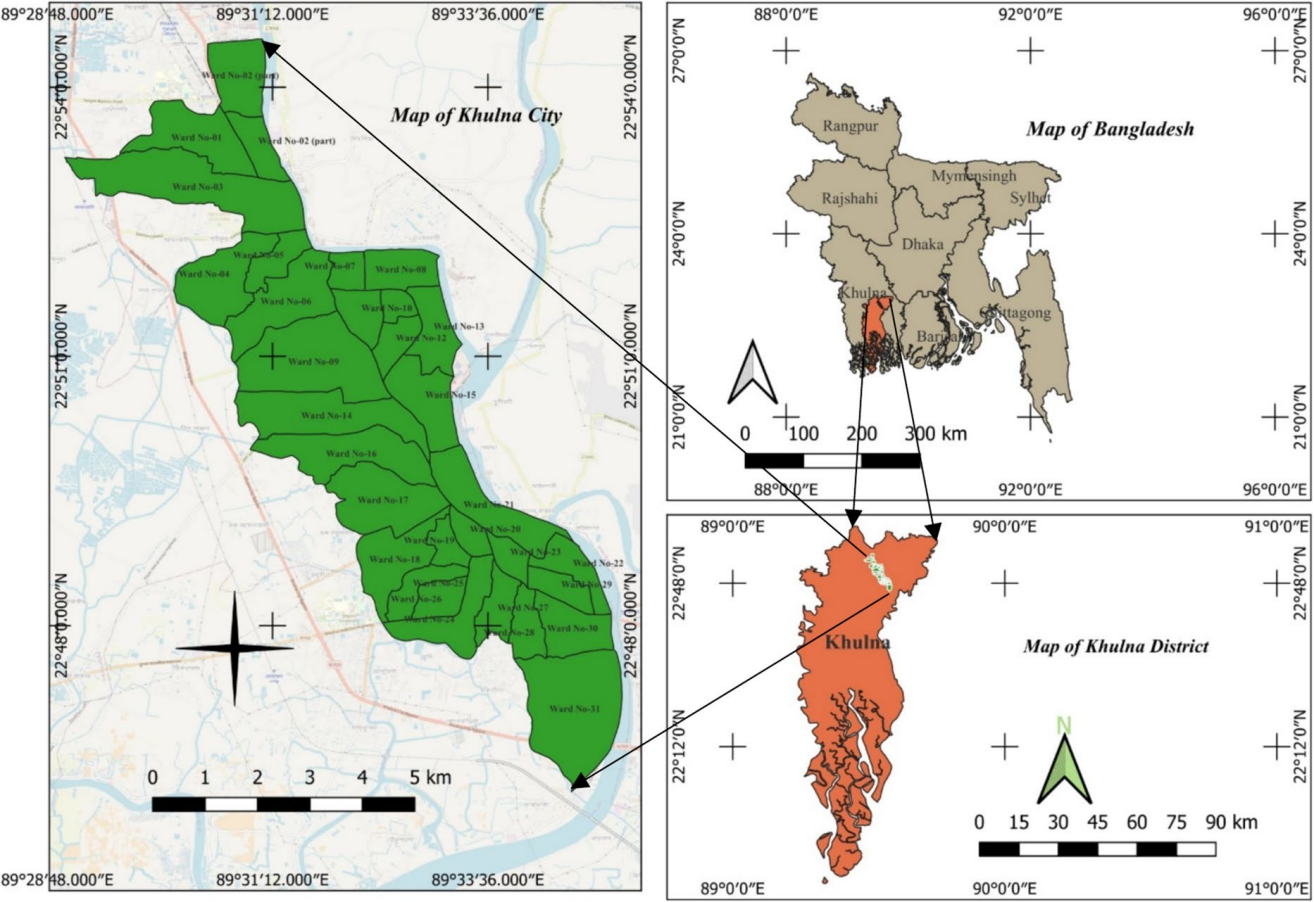
Map of Khulna City in Bangladesh

Khulna is the third largest city in Bangladesh and the gateway to the largest mangrove forest of Sundarbans. The city has an area of 45.65 km^2^ with a population of 1.5 million (Islam & Moniruzzaman, 2019). The city also has a proposed extension area of 99 km^2^, but the provision is still under consideration. Moreover, the city has also faced an initial surge of climate migrants during the last decade due to sea level rise and coastal erosion. There are floating populations around the city on a daily basis in search of livelihood (Rahaman et al., 2018). Khulna City is divided into 31 nos. municipal wards, as shown in Fig. 1. The conservancy department of Khulna City Corporation (KCC) is solely responsible for the solid waste management in Khulna City. The city area is on the banks of the Bhairab and Rupsha river. The city has mainly three landfill sites named Rajbandh, Soula, and Mathavanga, with an area of approximately 0.26 km^2^ (Rafew & Rafizul, 2022). The waste management sector still has no provision for a waste separation facility; however, the waste collectors carry separate sacks with their waste collection vans to separate plastics with values through surface screening of the collected waste. Therefore, the necessity of at least one waste separation facility and a reliable formal plastic recycling system is a top priority in order to improve the plastic waste management scenario of Khulna City. The map of Khulna City is shown in Fig. 1.

### Data Collection

**Fig. 2:**
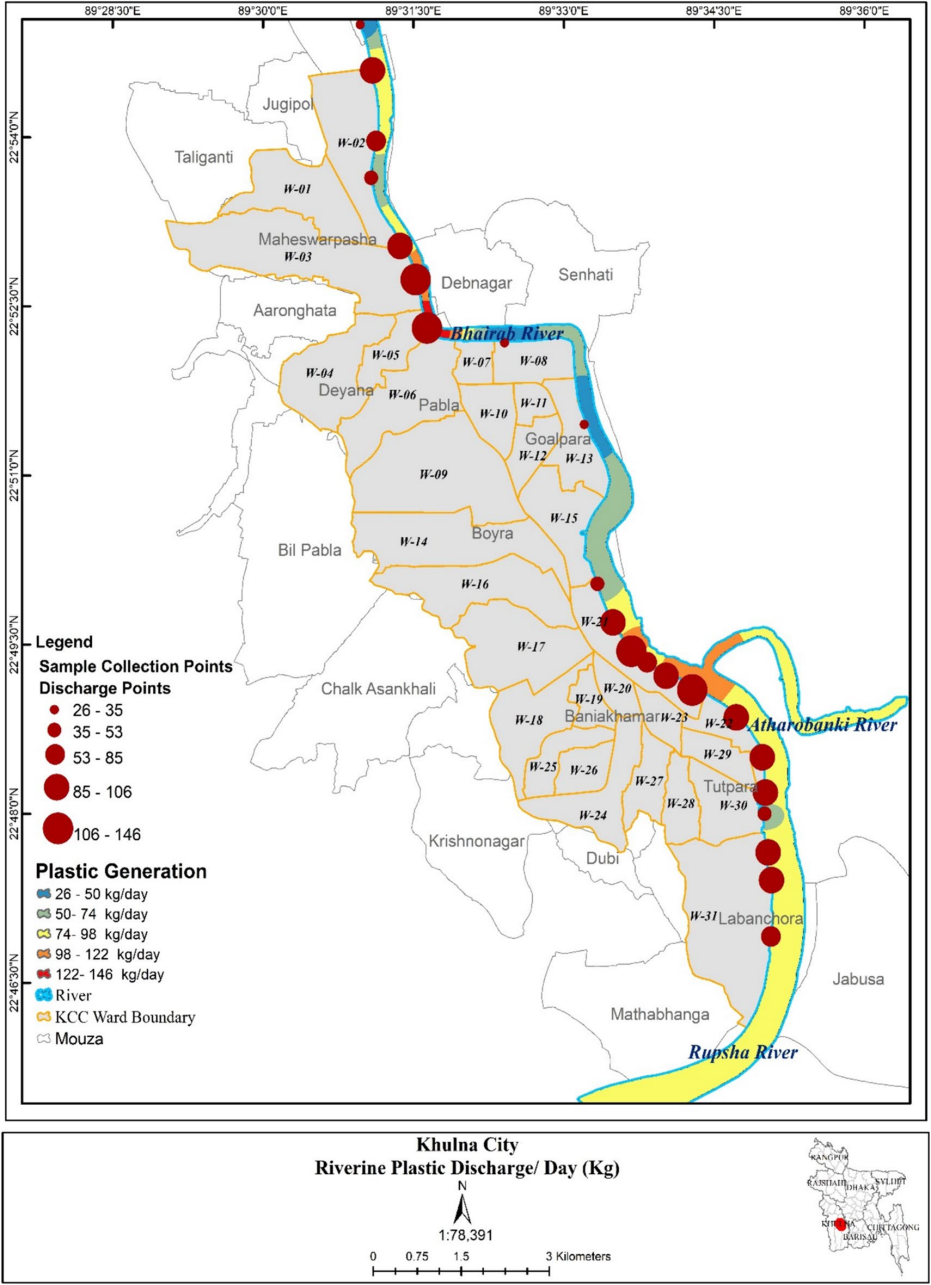
Riverine plastic discharge hotspot zones of Khulna City

In the first stage, primary data, along with a few secondary datasets for different sub-systems of the system dynamics model, has been collected and analyzed. In the case of this agent-based system dynamics simulation study for Khulna City, macro-scale factors such as regional GDP, plastic waste stock at landfill, income multiplier, total plastic waste generation, and riverine discharge of plastic waste, alongside numerous micro-scale factors such as riverine plastic discharge from each municipal ward of Khulna City, plastic generation per-capita, and annual turnover of each of the plastic recycling shop situated at Khulna, have been extensively considered with detailed dataset. The regional population and GDP data have been collected from the stated secondary sources of the Bangladesh Bureau of Statistics (2022), BBS (2022), and Sowgat and Roy (2020). For the acquisition of data for each municipal ward and with income level, a total of 615 nos. households, 10 nos. institutions, and 15 nos. commercial places with 75 nos. have been assessed. From municipal wards, waste generation of households and commercial places has been surveyed while taking 15 households of five different income groups from each of nine wards and nearby commercial places with their total waste as well as plastic waste production for the base year of 2023. The ward-wise plastic waste generation rate and its spatial distribution have been shown in the prospective ArcGIS map provided in Appendix C of the supplementary materials file denoted as Supplementary Fig. 1. The survey is being conducted in a close-ended format. Separate bins have been provided with instructions for each household, institution, and commercial place. Then the plastic waste from those bins has been collected with a seven days interval. The average municipal waste production of households has been found to be 0.48 kg per capita per day, with an average plastic percentage of 4.49 of the total generated waste (Noman et al., 2023). The survey was conducted while following practitioner guidelines for household waste analysis by Bidlingmaier, (2001) which recommends a 1% sample of the weekly generated waste is adequate to obtain a reasonably representative sample. In this study, a waste sample of approximately 1050 kg for 7 days has been collected from the stated 75 nos. locations, and the results are shown in Supplementary Table 1 in Appendix C. For the riverine discharge of the generated plastic in the Rupsha and Bhairab rivers, continuous assessment and macro plastic sample collection has been conducted and 25 potential plastic discharge locations near the river banks has been identified. For standard sampling procedure of macro plastics at riverbanks, a mixed transect method from European Commission by  González et al. (2016) of visual observation with collection and by Gasperi et al. (2014) with a manual collection of an approximate 2-kg subsample of the riverbank area with plastic debris in short the garbage patch. Riverbank plastics greater than 1.5 cm have been considered for quantification as macro-plastic in this study as per standardized specification for riverbank sampling protocol (Rech et al., 2014; UNEP, 2020). Five significant discharge sites have been selected based on the spatial location of commercial establishments such as market places, shops, and tourist points and a total of 10 kg plastic waste sample has been collected during the low tide with 5 m shore line transect on each site. A sample photograph of the riverbank solid plastic waste collection and recycle shop plastic stack is given in Appendix C as Supplementary Fig. 1 of the supplementary materials file. The sites were chosen not randomly but by obviously increased accumulation of waste, especially plastic waste. Samples were collected during the day over a two-week period at the end of summer and the beginning of the rainy season as per the standard described by Acha et al. (2003) and González et al. (2016). Most of the plastics found are single-use plastics like packets of chips, biscuits, food packaging, PET bottles, nets, plastic cups, and polybags. The acquired dataset has been incorporated in ArcGIS (version 10.8) to create a heat map based on the quantity of plastic found in the river banks during the assessment period with low tide for both upstream and downstream. From the assessment, the highest riverine plastic discharge has been observed at the Rupsha ghat area and Daulotpur Bazar ghat area, as shown in Fig. 2. A gross recycling plastics inflow to recycling shops for Khulna City has been found to be 27.70 tons per day during a direct survey of the 6-month period while assessing 35 nos. recycling shops mostly inside and around the city boundary. A gross total of 830.5 tons of recyclable plastic and 6.9 tons of non-recyclable plastic have been observed per month from the direct assessment of these shops. The gross turnover of all the recycle shops has been collected separately and the summed amount is around 715.806 million Bangladeshi Taka (BDT) for the base year with an average annual BDT of 20.45 million per shop per year (Saju et al., 2023, 2024). Only the recycle shops from the city periphery has been considered as they contribute to the plastic waste recycling outflow of the city. Data regarding plastic reuse has also been curated from Bari et al. (2012). The GIS location of the recycle shops assessed has been shown in Appendix C as Supplementary Fig. 2. The sample closed ended survey sheet for households and recycle shops has also been given in Appendix B of supplementary materials. The baseline source separation data has been assumed based on the household survey conducted. The public concern graphical dataset has been assumed based on the current mindset and practices of the general mass towards plastic pollution and riverine plastic pollution in Khulna City.

### Dynamic hypothesis for system dynamics model

**Fig. 3:**
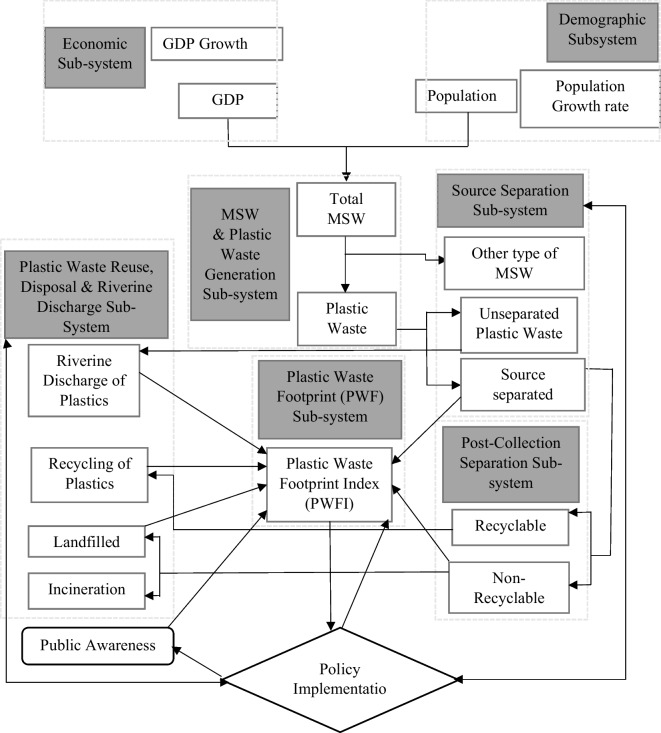
Flowchart for developing system dynamics model for plastic waste management of Khulna City

In order to define the system structure for navigating the system’s behavior in citation mode for this study, a casual-loop diagram or a conceptual model has been constructed as the dynamic hypothesis. System Dynamics (ASD) is widely recognized as a computer-oriented decision support tool, which not only incorporates the qualitative elements of the assessment of the results obtained but also has the applicability in analyzing the simulated processes of the potential plans, along with the consequences of the decision taken previously and during the simulation (Bala et al., 2017). A typical MSPW management system is influenced both hypothetically and practically by external and internal parameters. As discussed in the previous section, environmentally-extended input–output analysis (EEIOA) requires regional and transboundary datasets to accurately articulate the actual plastic waste flow scenario. Again, with respect to EEIOA, the overall system has been divided into several subsystems with micro-level agent-based parameters. Thus, economic and demographic aspects come into play. Also, as part of municipal solid waste generation, plastic collection and separation, post-collection and separation, treatment, disposal and transformation of plastic waste, and finally, the ecological footprint and public interest can be identified. Ecological footprint (EF) was first proposed by Rees (1992), which normally incorporates with the natural resource demand of humans and the quantity of waste it generates. A recent study by Mallick et al. (2021) has quantified plastic waste footprint (PWF) to investigate plastic reduction challenges and policy recommendations within the prism of sustainable development goal (SDG) during the pandemic scenario in India. The plastic waste footprint index has previously been quantified by Liu et al. (2023) while considering the plastic material footprints of plastic usage and plastic waste footprint in China. The above-stated study by Liu et al. (2023) is one pioneering study on the plastic waste footprint index that not only has incorporated scenario analysis (baseline and policy impacts) but also has addressed the life-cycle-based plastic environmental footprint of enterprises involved in plastic packaging. Similarly, for this study, a plastic waste footprint index (PWFI) has been quantified while assessing the plastic waste flow around the city. An increasing index value denotes the deterioration of the overall plastic management situation, while a lower PWFI value represents a more managed scenario of plastic waste flow in the city. The index values further are required to inform the policy makers about the current situation alongside guiding the concerned authorities on setting up specific goals towards plastic waste reduction pathways (Liu et al., 2023). The practical implication of thus index values will not only give a perception to the general mass regarding the ever-present yet subconsciously ignored plastic materials and the waste being produced from these materials but also will set standard margins for denoting the safe, medium, and extreme conditions of plastic pollution in a region, somewhat similar to air quality index or ultra violet (UV) index values. In addition, policies to reduce plastic waste flows, discharge of plastics into rivers, and promote recycling, reuse, and conversion of plastics will be developed to promote sustainable practices and reduce the environmental impact of plastic waste. Furthermore, both the generic hypothetical and specific factors used in the system dynamics model have implication beyond a specific region or country. Though microplastic (< 5 mm) is a pressing issue in our present world as it originates from the degradation of macroplastic (> 5 mm) mostly (Strokal et al., 2023), they are not best suited for PWFI calculation due to their unique characteristics. However, before quantifying the amount of microplastic, it is evident that to conduct macro plastic waste quantification and life cycle analysis, which a whole different level of research itself. Rather than measuring all the pollutants such as particulate matter, airborne microplastic, carcinogens, industrial waste, and volatile organic components, the PWFI in this study is concentrated specifically visible municipal macro plastic both inland and riverine (> 1.5 cm) waste as per (UNEP, 2020) protocol discussed before, specifically for determining plastic flow prevention policy measures. Apart from that, airborne plastic particles are fragments of microplastic or mostly nano-plastics that originates from industrial processes like tire abrasion and synthetic fiber shredding (Tan et al., 2024), while this study emphasizes municipal solid plastic waste flow and prevention policy. The inclusion of such small-scale micro- and nano-plastic particles is less relevant given to the purpose of the study. Moreover, including microplastic and nano plastic in a single platform with macro plastic will create complexity regarding the implication of policies and thus will deviate attention from more pressing issues regarding traditional plastic waste management and prevention (Das et al., 2021; Nelms et al., 2022). Furthermore, plastic buried underneath the ground is difficult to identify and has an indirect impact (Li et al., 2023) if compared to the visible macro plastic. Similar to the above-stated reason, PWFI mainly focuses on measurable forms of plastic in order to focus on prevention and mitigation efforts (Guillotreau et al., 2023). Though the inclusion of this parameter is important, focusing on visible and measurable forms of plastic pollution is far more important for footprint calculation and management strategies. From the geographic or spatial perception, the developed ASD model and its subsystem for this study have unambiguity in terms of application. However, for the mountainous region, the inclusion and exclusion of modified factors for plastic waste management might be necessary (Semernya et al., 2016). The diagrammatic illustration of the initially hypothesized development of the modelling process is shown in Fig. 3.

### Casual-loop diagram

The primary structural element within a system’s parameter covering the key variables inside an endogenous boundary is the purpose of the development of a casual loop diagram. A casual loop is the polarized illustration of the feedback loops and is shaped in the control theory block diagrammatic form (Bala et al., 2017). For this study, the causal loop diagram has been developed using iThink STELLA Licensed version 1.9.4 software. Stella is a powerful tool for addressing multi-varieties of complex system parameterization, scenario, and sensitivity analysis with altered parameters based on real-world datasets, visualized feedback loop-based programming, simulation, and analysis with iterative model refinement features. This not only allows the model’s accuracy with continuous improvement but also incorporates practical limitations through iterative experiments (Hargrove, 2003; Lindfield, 1992). Waste and plastic waste generation and management require addressing complex inter-connected parameters with non-identical unit values due to their multi-dimensional nature. This is where Stella software plays a significant role in ever-evolving understanding as well as empirical observations in plastic waste management and policy simulation.

The casual loop diagram (CLD) has been developed as a theoretical framework for analyzing municipal solid waste generation, plastic waste generation, plastic waste separation, recycling, riverine plastic discharge, public perception, and its effect on reducing plastic waste generation. The reinforcing loops and balancing loops are denoted by the color code in the CLD. A higher value of the plastic waste footprint index (PWFI) denotes a faulty plastic waste management scenario along with a high level of riverine plastic discharge in the city area, and it naturally will give rise to public concern. In this study, PWFI is computed using the following Eq. (1).

1 $$PWFI=({w}_{1}\times RCP+{w}_{2}\times NRCP+{w}_{3}\times PNC+{w}_{4}\times PSL+{w}_{5}\times RPL-{w}_{6}\times CP)\times PoR$$

**Fig. 4:**
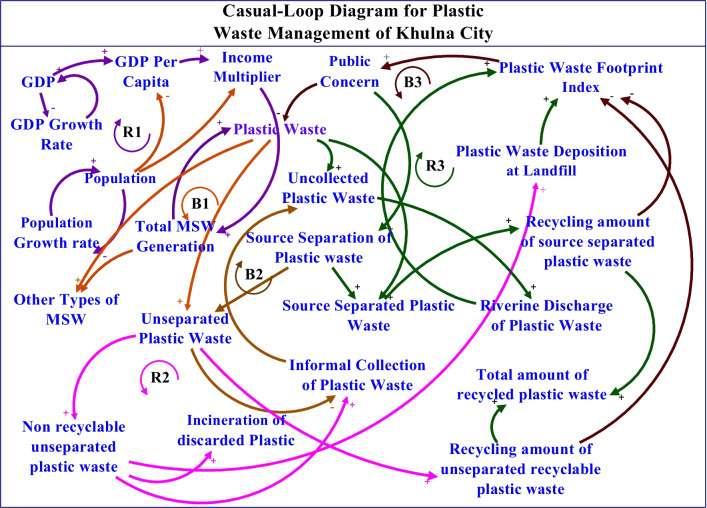
Casual-loop diagram for plastic waste management of Khulna City

Here, RCP is the ratio of the timestamp value of a recyclable portion of the collected plastic to its baseline value, NRCP is the ratio of the timestamp value of the recyclable portion of the collected plastic with respect to the base value, PNC is the timestamp value of plastic with no collection facility to the base value, PSL is the ratio of time stamp value for plastic waste stock at the landfill to its base value, RPL is the ratio of time stamp value for riverine plastic waste discharge to its base riverine plastic discharge value, CP is the ratio of the time stamp value of conversion of plastic waste to it base potential quantity, and PoR is the timestamp ratio of population to the base population. The weighted average factors have been denoted as w_1_, w_2_, w_3_, w_4,_ w_5_, and w_6_, with an average weighted value of 0.5 for each. The casual loop diagram of the hypothesized municipal solid plastic waste (MSPW) management scenario of Khulna City has been shown in Fig. 4.

### Stock-flow diagram

A stock-flow diagram is the physical structure of a system usually representing a detailed illustration of CLD. The actual state of the independent variables that have been considered in the ASD model is represented with stock for the plastic waste management scenario of Khulna City. In this built model, flow changes dynamically with respect to the condition of the system and decisions. Control theory block diagram represents the differential equations that have been articulated within a system’s boundary (Bala et al., 2017). According to Sterman (2000), “The stock and flow diagram has a precise and unambiguous mathematical meaning. Stocks accumulate or integrate their flows, the net flow into the stock is the rate of change of the stock.” Stocks can be represented as an integral equation represented through Eq. (2) or first-order finite differential equation as of Eq. (3).

2 $$stock\left(t\right)=stock\left(t_0\right)+\int_{t_o}^t\left(\mathrm{inflow}\left(\mathrm t\right)-\mathrm{outflow}\left(\mathrm t\right)\right)\mathrm{dt}$$

Here, inflow (t) has been represented at any time in-between the present time t and initial time t_0_. Equivalently, if the inflow is less than the outflow, then it represents the stock’s net rate of change and thus defining the differential equation as given in Eq. (3).

3 $$\frac{d(stock)}{dt}=inflow \left(t\right)-outflow(t)$$

**Fig. 5:**
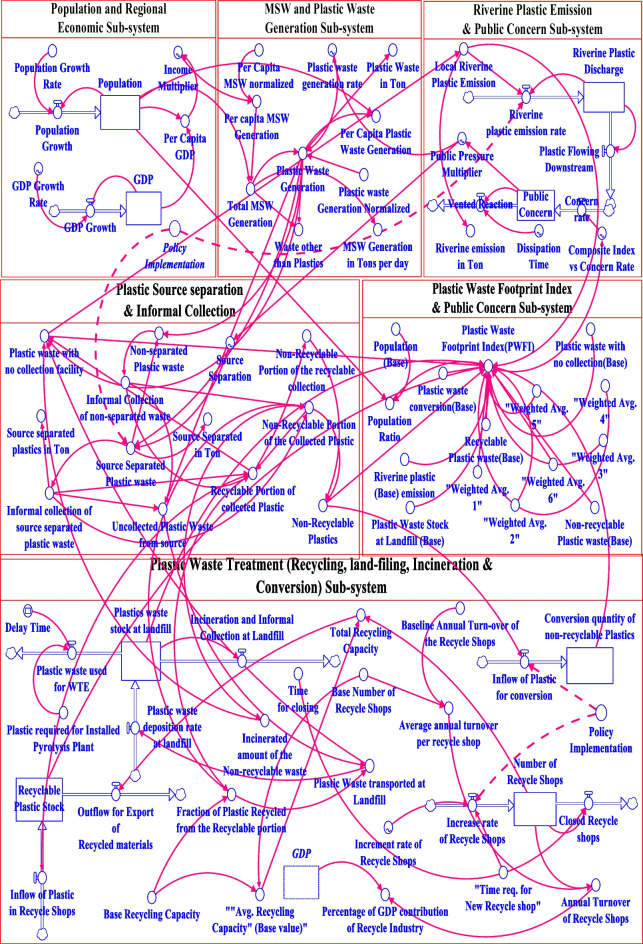
Stock-flow diagram of the municipal plastic waste flow of Khulna City

Stella software generally consider three consecutive methods for solving these initial value problems. These methods are (a) Euler’s method, (b) second-order Runge–Kutta method, and (c) fourth-order Runge–Kutta method. Specifically for this study, Euler’s integration method has been used. Particularly for this study, a special computational procedure for function without integration has been used for smooth operation of the system. These functions are responsible for the alteration of the instantaneous amplitude of signals passing through the procedural integration of the built system. Moreover, for this study, exponential, logarithmic, and linear interpolation functions have been constructed due to the mostly non-linear inter relation of the input dataset. The model has been divided into six sectors, as discussed before, and is shown in Fig. 5. For each of the sectors, the quantum factors (micro dataset) together act as agent-based while the accumulated stock parameters alongside factors including vast interpolated and regression dataset work together as marco parameters to develop a knit system. A number of fundamental equations have been considered while constructing the stock-flow model of plastic waste footprint for Khulna City. Moreover, there is no formal waste recycling or treatment facility available in the city area in the baseline scenario. So most of the plastic separation and collection has been dependent on the informal procedure. The developed ASD model however considers all the relevant factors regarding plastic waste flow, source separation, collection, recycling, riverine discharge, etc. The plastic incineration, pyrolysis practice, has also been considered in the ASD model due to the existing baseline scenario, as stated in the “Data Collection” section.

To be precise, the stock-flow model is a detailed representation of the CLD model that has been developed by a detailed study of plastic flow around Khulna City. PWFI has been calculated while considering the factors of recyclable and non-recyclable plastic waste, net riverine plastic flow, and potential plastic recycling facilities, as well as plastic waste stock at landfills throughout the simulation time period. All the parameters have been considered for quantifying PWFI in this study; however, the characterized recycling values such as PET, HDPE, or LDPE have not been considered due to the unavailability of a precise detailed investigation of macroplastic particle flow to the recycle shops. With the increased value of PWFI, public concern will increase, and an increased public concern will raise the awareness of the citizens and thus will play a vital role in reducing municipal plastic waste generation. Some of the equations of the built stock-flow model are shown below. The full set of codes and equations is given in Appendix A of the supplementary file.

4 $$''\mathrm{Informal}\_\mathrm{Collection}\_\mathrm{of}\_\mathrm{non}-\mathrm{separated}\_\mathrm{waste}''=\mathrm{DELAY}\left(\left(''\mathrm{Non}-\mathrm{separated_Plastic_waste}''\ast0.10\right),1.2\right)$$

5 $${\text{Riverine}}\_{\text{Plastic}}\_{\text{Discharge}}\_{\text{Accumulation}}({\text{t}}) =\mathrm{ Riverine}\_{\text{Plastic}}\_{\text{Discharge}}\_{\text{Accumulation}}(\mathrm{t }-\mathrm{ dt}) + ({\text{Local}}\_{\text{riverine}}\_{\text{plastic}}\_{\text{emission}}\_\mathrm{rate }-\mathrm{ Plastic}\_{\text{Flowing}}\_{\text{Downstream}}) *\mathrm{ dt}$$

6 $$\mathrm{Non}\;-\;\mathrm{separate_Plastic_waste}''=\mathrm{IF}(\left(\mathrm{Plastic_Waste_Generation}-\mathrm{Source_Separated_Plastic_waste}\right)\;<\;\left(\mathrm{Plastic_Waste_Generation}\right)\mathrm{THEN}\left(\mathrm{Plastic_Waste_Generation}-\mathrm{Source_Separated_Plastic_waste}\right)\mathrm{ELSE}\left(0\right)$$

7 $$''\mathrm{Plastic}\_\mathrm{Waste}\_\mathrm{Footprint}\_\mathrm{Index}(\mathrm{PWFI})''=((''\mathrm{Weighted}\_\mathrm{Avg}.\_1''\ast(\mathrm{Recyclable}\_\mathrm{Plastic}\_\mathrm{Stock}/''\mathrm{Recyclable}\_\mathrm{Plastic}\_\mathrm{waste}(\mathrm{Base})''))+(''\mathrm{Weighted}\_\mathrm{Avg}.\_2''\ast(''\mathrm{Non}-\mathrm{Recyclable}\_\mathrm{Plastics}''/''\mathrm{Non}-\mathrm{recyclable}\_\mathrm{Plastic}\_\mathrm{waste}(\mathrm{Base})''))+(''\mathrm{Weighted}\_\mathrm{Avg}.\_3''\ast(\mathrm{Plastic}\_\mathrm{waste}\_\mathrm{with}\_\mathrm{no}\_\mathrm{collection}\_\mathrm{facility}/''\mathrm{Plastic}\_\mathrm{waste}\_\mathrm{with}\_\mathrm{no}\_\mathrm{collection}(\mathrm{Base})''))+(''\mathrm{Weighted}\_\mathrm{Avg}.\_4''\ast(\mathrm{Plastics}\_\mathrm{waste}\_\mathrm{stock}\_\mathrm{at}\_\mathrm{landfill}/''\mathrm{Plastic}\_\mathrm{Waste}\_\mathrm{Stock}\_\mathrm{at}\_\mathrm{Landfill}\_(\mathrm{Base})''))+''\mathrm{Weighted}\_\mathrm{Avg}.\_5''\ast(\mathrm{Local}\_\mathrm{Riverine}\_\mathrm{Plastic}\_\mathrm{Emission}/''\mathrm{Riverine}\_\mathrm{plastic}\_(\mathrm{Base})\_\mathrm{emission}'')-(''\mathrm{Weighted}\_\mathrm{Avg}.\_6''\ast(''\mathrm{Conversion}\_\mathrm{quantity}\_\mathrm{of}\_\mathrm{non}-\mathrm{recyclable}\_\mathrm{Plastics}''/''\mathrm{Plastic}\_\mathrm{waste}\_\mathrm{conversion}(\mathrm{Base})'')))\ast\mathrm{Population}\_\mathrm{Ratio}$$

The plastic waste footprint index (PWFI) has been calculated by dividing each simulated parameter value by its base value and multiplying it by the respective weighted average. Then, all the parameters are either added or subtracted as per the polarity it imposes. The delay function is used to indicate the time delay that occurred for collecting a certain quantity of plastic, and it is identified while conducting the on-field survey. Also, the if–then-else function has been used in this ASD model to indicate certain conditions that may occur. The in-root ASD model has 86 variables (array expansion in parens). It is an in-root model and 0 additional modules with 6 sectors. The number of stocks (8), flow (14), converters (63), constants (26), Eqs. (52), and graphical (8) have been used for developing the ASD model.

## Policy analysis

Experimenting with the existing system would be impractical from both socioeconomic and time consumption perspectives. Furthermore, while experimenting with different pathways, it is almost impossible to turn back to the baseline situation if the policy measure performs unexpectedly. To overcome this phenomenon, system dynamics simulation of policy measures can be a vital tool for assessing the effects and flaws of proposed policy measures (Bala et al., 2017). The objective of this section is to evaluate the existing and future policy options regarding plastic waste management in Khulna City.

In order to get a perception about the altered policy options, the two subsequent policies have been considered in this study while keeping in mind the aggravated effect of plastic pollution on both inland and marine environments as well as the century-long footprint it will be leaving alongside. The two policies are denoted as policy 1 and policy 2, and the parameter is considered for these two improvised policy scenarios.

- *Policy 1*: An increase in the rate of recycle shops has been considered an average of 2 nos./year from a base scenario of zero increment as there is a lack of local government incentives in this sector. The source separation percentage has been increased to 80% from a baseline scenario value of 63%. The conversion quantity of non-recyclable plastic waste has been considered as 20% of the total generated non-recyclable quantity. Though such technology has not yet been established in the country apart from very few plastic manufacture factories, from a circular economic perspective, this is the most prominent way to reduce the plastic waste footprint in this coastal city. Finally, a mere 20% reduction of the local riverine plastic emission has been considered, which can be achieved by providing a frequent number of bins at regular intervals to the identified hotspot zones contributing to riverine plastic discharge alongside awareness and collection facilities.
- *Policy 2*: This policy has been considered after observing both knit improvement as well as the downfall of the expected outcome during the simulation period from 2023 to 2040. For this policy, the rate of plastic recycling shops has been rate increased to an average of 3 nos. per year with their average recycling capacity. The conversion quantity of non-recyclable plastic waste has been raised to 69%, which indicates a technological advancement in this era of the fourth industrial revolution for Bangladesh is eminent for sustainable plastic management and sustainable development in the long run. The proportion of separate collection is set at a maximum of 80%, as complete separation at source is most difficult to achieve in Bangladesh. Finally, identical to “policy 1,” a 50% reduction in plastic discharges to rivers was assumed.

## Results and Discussion

In this study, the MSW generation, plastic waste generation, deposition, and riverine plastic discharge have been predicted to get the futuristic perception of plastic waste flow in the city. The simulation results have been generated while considering Euler’s integration method and are illustrated in the manuscript through multi-scale comparative graphs. The later sections emphasized on the reduction of the plastic flow while emphasizing on plastic source separation, collection as well as innovations to reduce plastic waste around the city area through consecutive policy simulations. The numerical simulation results of important parameters of the baseline scenario are shown in Table 1. Additionally, sector-wise results of the important parameters are represented graphically in the following sections.

**Table 1:** Baseline simulation results for plastic waste generation of Khulna City from 2023 until 2040

Year	Population (million)	MSW generation (ton/day)	Plastic waste generation(ton/year)	Per capita plastic waste generation (kg)	Recyclable portion of collected plastic (kg)	Local riverine plastic emission (kg)	Plastic waste footprint index (PWFI)
2023	1.5	703	13×10^3^	8.92	7.8*10^6^	512×10^3^	5.57
2024	1.51	720	13.6×10^3^	8.96	7.9*10^6^	529×10^3^	5.56
2025	1.53	738	13.8×10^3^	9.03	8.04×10^6^	545*×0^3^	5.72
2026	1.54	757	14.1 ×10^3^	9.15	8.22×10^6^	560×10^3^	6.03
2027	1.56	777	14.×*10^3^	9.28	8.42×10^6^	576×10^3^	6.47
2028	1.57	799	14.8×10^3^	9.44	8.64×10^6^	593×10^3^	7.04
2029	1.59	822	15.2×10^3^	9.6	8.86×10^6^	609× 10^3^	7.74
2030	1.6	846	15.6×10^3^	9.73	9.08×10^6^	621×10^3^	8.54
2031	1.62	872	15.9×10^3^	9.86	9.28×10^6^	634×10^3^	9.47
2032	1.63	898	16.3×10^3^	10	9.5 ×10^6^	650×10^3^	10.5
2033	1.65	926	16.7×10^3^	10.1	9.7×10^6^	661×10^3^	11.6
2034	1.66	956	17×10^3^	10.2	9.87×10^6^	669×10^3^	12.8
2035	1.68	988	17.3×10^3^	10.3	10.1×10^6^	686×10^3^	14.2
2036	1.69	1.02×10^3^	17.5×10^3^	10.4	10.2×10^6^	687×10^3^	15.8
2037	1.71	1.06×10^3^	18.2×10^3^	10.7	10.6×10^6^	739×10^3^	17.6
2038	1.73	1.09×10^3^	19×10^3^	11	11×10^6^	774×10^3^	19.5
2039	1.74	1.13×10^3^	19.7×10^3^	11.3	11.5×10^6^	803×10^3^	21.6
2040	1.76	1.18×10^3^	20.5×10^3^	11.6	11.9×10^6^	834×10^3^	24

### Population, GDP, MSW generation, and plastic waste generation

**Fig. 6:**
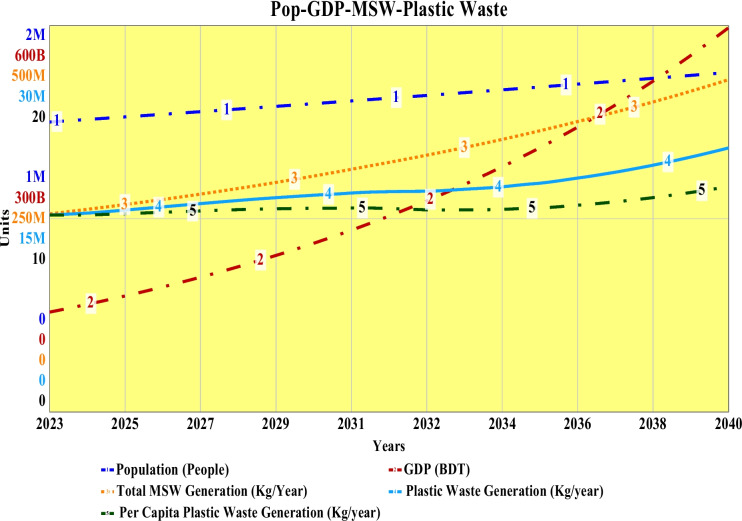
Population, GDP, MSW generation, plastic waste generation, and per capita plastic waste generation of Khulna City from 2023 to 2040

The simulated projection of population, GDP, per capita GDP, MSW generation, per capita MSW generation, and plastic waste generation for Khulna City is shown in Fig. 6. The city has a population of about 1.5 million and by 2040, it has shown a linear increase to 1.76 million. The regional GDP for Khulna has been 155 billion BDT, which exponentially increases to 597 billion BDT by 2040. Also, per capita GDP represents a similar pattern with an ultimate per capita annual income of 339 thousand BDT by 2040. The quantity of MSW generation and per capita MSW generation show a linear increase from a baseline value of 703 tons per day and 171 kg to 799 tons per day and 186 kg by 2028, respectively. Then it starts to increase exponentially, and by 2040, total MSW and per capita MSW generation will become 1.18 thousand tons per day and 245 kg respectively. The plastic waste generation per capita for the baseline year of 2023 has been 8.92 kg, which is quite identical to the recently published World Bank report by Yoshijima et al. (2021) of 9 kg. The total plastic waste for Khulna City for the baseline has been 13.4 thousand tons. Both the per capita plastic waste and total plastic waste show an initial linear increase until 2036 and then start to increase exponentially to an ultimate quantity of 11.6 kg and 20.5 thousand tons respectively by 2040. These results represent a clear indication of the necessity of formal policy requirements to minimize plastic waste generation. Though the per capita plastic waste consumption of Bangladesh is quite less compared to neighboring countries like India and Myanmar, but the country’s plastic waste generation is problematic due to the absence of formal waste management facilities and technologies at the regional level. It is imperative that the concerned authorities and policy makers should emphasize reducing plastic waste generation and thus take advanced initiatives to improve the scenario.

### Plastic waste source separation, collection, landfilling, and riverine discharge

**Fig. 7:**
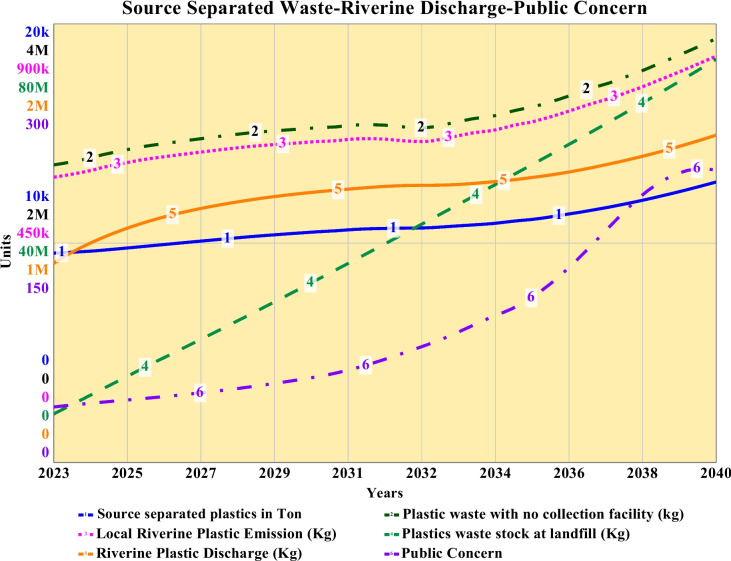
Plastic source separation, recyclable plastic, plastic with no collection facility, and local riverine plastic discharge and accumulation of plastic waste for a period from 2023 to 2040

The source-separated quantity of plastic waste for 2023 has been 8.35 thousand tons, which increased in a mixed linear and slight exponential pattern to a final value of 12.8 thousand tons by the end of 2040. The exponential rise is due to the exponential increase in plastic waste generation, as shown in Fig. 6. The baseline source separating is done in an informal way during the collection phase. Also, most of the source separation initiatives are being conducted informally by feriwalas, housemaids, waste pickers, etc., who collect or buy reusable plastics from households, transfer stations, and local undesignated dumpsites. During the survey period, we have observed that this value chain has increased over the past couple of years. As a result, despite less or no plastic separation at household and community levels, the source-separated quantity of plastic increased to 62.4% of the total generated plastic by the end of 2040. Apart from the delayed collection and other sorts of non-conventional plastic mitigation measures such as open burning, plastic with no collection facility increased logarithmically until 2033 to a value of 3.06 million kg from a base value of 2.37 million kg, and later on, it increased linearly to 3.87 million kg by 2040. Riverine plastic discharge has been a significant discovery for this study, and the quantity initially increases exponentially until 2033 to 661 thousand kg from a base discharge quantity of 512 thousand kg in 2023, and from 2034, it starts to increase exponentially to an ultimate riverine discharge quantity of 834 thousand kg by 2040 with the existing baseline practices. Accumulation of riverine plastic waste shows an initial logarithmic and later stage exponential increase from a base value of 908 thousand kg in 2023 to an ultimate accumulation of 1.49 million kg by 2040. Plastic waste stock in landfills shows a linear increase due to the usual routine practice of collecting and transporting it to dumping stations, and the quantity has been from 8.85 million kg in 2023 to a piled up 69 million kg of plastics in 2040. For curve-line 1, 2, 3, and 4, the slight exponential rise is due to the exponential increase of plastic waste generation, as shown in Fig. 6 previously. In brief, this trend is also identical to total plastic waste generation for this baseline simulation. More plastic pile landfills mean less area for waste, which will severely decrease the lifespan of the landfill. The combination of all such phenomena has a looped connection with public concern as it starts to increase exponentially until 2038 and then represents a logarithmic increase from a mere baseline value of 38 during 2023 to 168 by 2040. A lagged awareness-response among inhabitants rises due to a lack of knowledge-based realization or eventually due to a lack of willingness to regard the issue among inhabitants alongside an ill-practiced tradition of transferring responsibilities. It shows how the situation will degrade over the course of time from a present moderate level of concern. The graphical representation of these results is shown in Fig. 7.

### Plastic waste footprint index, recyclable portion of collected plastic, non-recyclable plastic, regional plastic industry’s contribution to society, and fraction of plastic waste recycled

The plastic waste footprint index (PWFI) is a demarcation of the deterioration or improvement of the footprint of plastic waste in the adjacent environment. An initial value of 5.57 for PWFI for the baseline scenario represents the potential upcoming surge of plastic waste around the city area while considering numerous adjacent parameters. The PWFI increases exponentially throughout the simulation period. Until 2032, PWFI has been below 10, but after 2032, it kept increasing drastically to a final footprint index value of 24 by 2040. The simulated combination of all the parameters has been normalized and converted to this single-point indexed value. Each of the parameters has a significant effect on this indexed value, as shown in the CLD developed. Due to the extensive, detailed, and elaborate nature of the dataset, the author has chosen to discuss summarized aspects of the resulting dataset of the simulation. A sample of the simulation event with resulted graphs at time step 2038.25 has also been provided in the supplementary material file as supplementary Fig. 3.

**Fig. 8:**
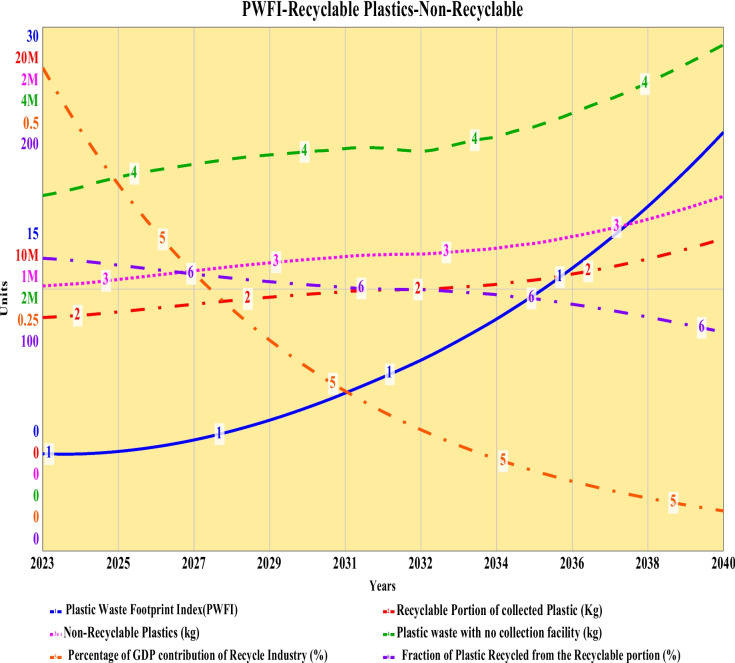
Plastic waste footprint index, public concern, plastic waste stock at landfill and non-separated as well as collected plastic waste for Khulna City from 2023 until 2040

A recyclable portion of the collected plastic increases slightly exponentially until 2034 to 10.1 million kg from a base quantity of 8.91 million kg. After that, growth becomes slightly exponential to an ultimate quantity of recyclable plastic of 12 million kg by 2040. The non-recyclable portion of the collected plastics increase patterns shows a slight exponential increase until 2032 to 1.13 million kg from a base quantity of 1.01 million kg. Then, it started increasing slightly exponentially until the end of the simulation period to a final value of 1.35 million kg. The plastic recycling export industry has been considered a “thrust sector” with the highest priority in Bangladesh “Export Policy 2018–2021.” Presently, the contribution of plastic products is estimated to be 1% of the GDP, with as many as 5000 firms around the country operating in the sector, while 98% of it is small and medium-sized firms (Razzaque et al., 2020; Siddique et al., 2022). The baseline simulation shows that the existing export quantity of medium- and small-scale recycling industry of Khulna contribute to 0.461% of the regional GDP during 2023. As discussed in the data collection section, a handful number of medium and small-scale recycle shops are located outside the city boundary, which trade recycled pellets to the large-scale recycle shops that are partnered with export processing Zones (EPZ) around the country. While assessing, it has been found that in Khulna City, a gross quantity of 830 tons of plastic waste per month is being recycled and exported to EPZ by the chain of 35 recycling shops during the baseline year of 2023. The recyclable portion indicates the recyclable plastic waste generated inside the city boundary, while the fraction of plastic recycled from the recyclable portion represents the recycled and exported quantity of plastic from the 35 nos. shops located both inside and at the periphery of the city corporation boundary. Due to this reason, for the baseline, the fraction of plastic recycled from the recyclable portion has been initially 112% for the baseline year of 2023 and declines slightly logarithmically to a value of 83.5% by 2040. Finally, for the baseline scenario, there have been primary concerns regarding the plastic waste and deposition around the secondary disposal sites, riverbanks, bus and train stations of the city, but little or no formal implementation has been observed to mitigate the plastic waste generation of the city area. Most of the non-separated plastics have been single-use plastic, to be precise, polythene bags, food wraps, etc. The simulated results for the baseline scenario have been shown in Fig. 8.

### Sensitivity analysis results of the ASD model

The purpose of the sensitivity analysis is to compile or validate the model structures and associated input dataset of the system dynamics model for boundary adequacy in extreme conditions (Smith et al., 2008). As discussed in the methodology section, 1000 nos. sensitivity runs have been considered for representing comparative graphs through the sensitivity analysis process. Sensitivity parameter values have been distributed uniformly based on regional datasets acquired from the base run and secondary data sources. Detailed data analytical tables and combinations with output parameters for thus 1000 sensitivity analysis, confidence intervals, histograms, and comparative graphs can be visualized in the Stella interface window for each graph separately during simulation. However, due to the extensive vast nature of this dataset, they have been stored in data cloud respiratory, which can be made available upon request. The sensitivity graph for the 1000 nos. runs, confidence graph for identifying the frequency of most number of runs in a region, and histogram representing the value range of those runs for municipal plastic waste management of Khulna City are illustrated in the following section.

#### Sensitivity for plastic waste generation in Khulna City

**Fig. 9:**
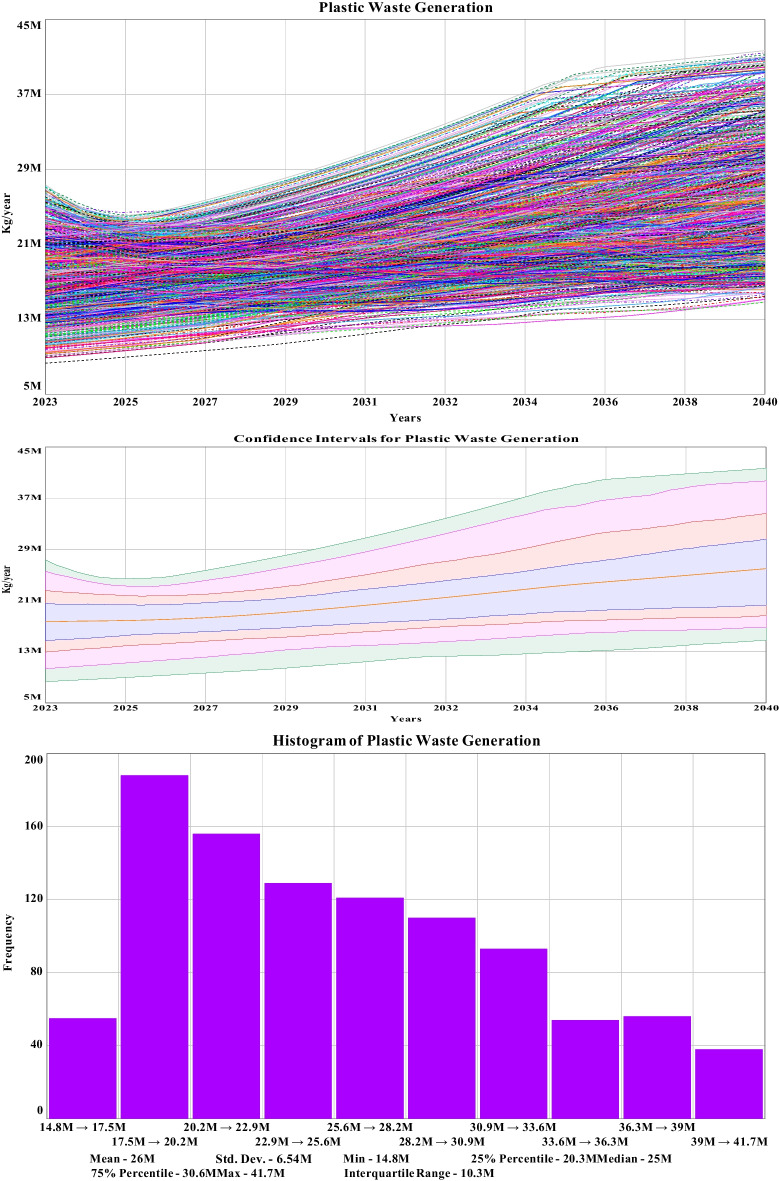
Sensitivity runs, confidence interval, and histogram for plastic waste generation of Khulna City until 2040

**Fig. 10:**
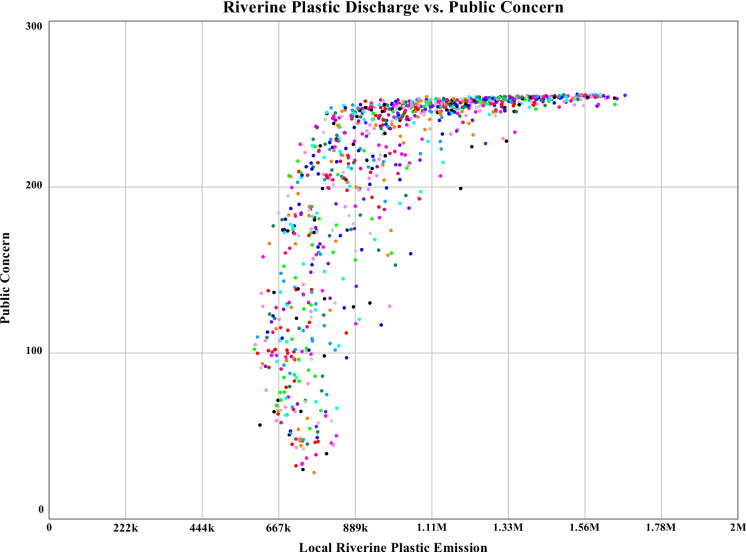
Scatter graph for riverine plastic waste discharge against public concern from 2023 to 2040

The sensitivity analysis results after the alteration of sensitive parameters have been simulated with 1000 consecutive runs. However, it is hard to distinguish data for a particular run from the produced sensitivity diagram of plastic waste generation. To overcome this limitation, confidence interval graphs have been considered. The confidence interval graph in this section for plastic waste generation indicates that 50% of the runs fall in between the range of 13.6 to 22.7 million kg per year with a mean of 18.2 million kg. For slicing through the confidence interval graph, histograms have essentially been considered. In the histogram, 190 out of 1000 runs fall in the region of 17.5 to 20.2 million kg per year. The maximum, minimum, and mean values of plastic waste generation have been observed to be 41.7 million kg, 14.8 million kg, and 26 million kg respectively. The simulated dataset has been divided into four equal parts to observe the interquartile range (IQR) of 10.7 million kg, and the standard deviation has been observed well below the IQR of 6.54 million kg, indicating not only the controlled clustering of the dataset around the mean value but also the precise reliability of the base model. The simulated graphs for the above-stated section are shown in Fig. 9. The comparative scattered graph between plastic waste generation and public concern for sensitivity run is shown in Fig. 10. Starting from a flat surface, the scattered graph gets uniformly distributed over time while reducing the mean plastic waste generation with public concern through the simulation period. The Sobol sequence sampling during the sensitivity run was selected to get a more organized scattered pattern for the dataset. It has been observed that through the progression of time, the public concern increased to the highest value of 255.96 from the lowest value of 16.67 due to the uniform distribution of the sensitive parameters. The plastic waste generation has reduced to a minimum value of 663 thousand kg at run 553 to the highest value of 158 thousand kg. The goal of this comparative graph is to find the boundary adequacy for the simulation, and analyzing the data might provide the best-suited combination for a sustainable plastic management feature. It is a form of data optimization with altered parameter values. These datasets mainly represent the output results of numerous combinations during the sensitivity simulation.

#### Sensitivity of PWFI

**Fig. 11:**
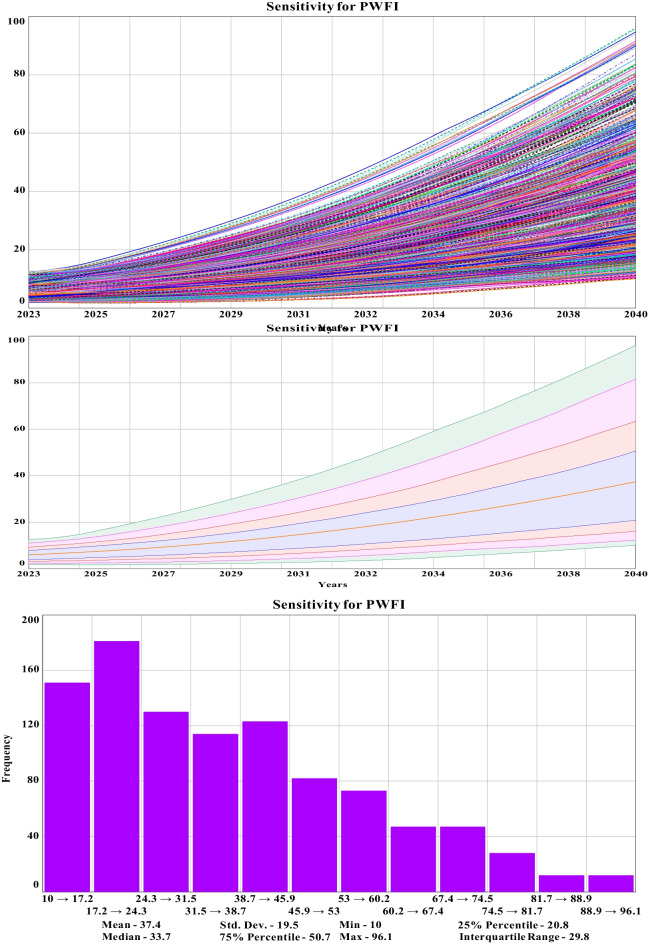
Sensitivity runs, confidence interval, and histogram for plastic waste footprint index until 2040

**Fig. 12:**
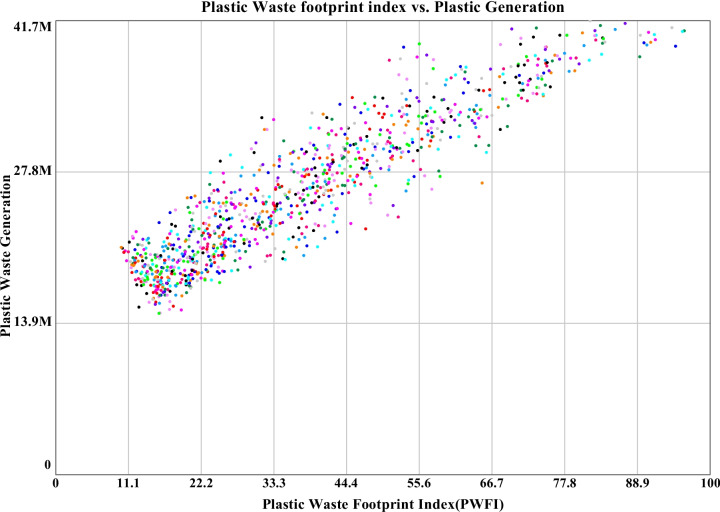
Scatter graph for PWFI against plastic waste generation from 2023 to 2040

PWFI is a representation of the plastic waste footprint index for this study, and it is proportional to the sonority value of the simulation. An uncontrolled value is an indication of deteriorating environmental quality, while a lower value indicates the sustainability tiers of the system. In brief, from the confidence interval graph, it has been observed that 50% of the PWFI value falls in between the range of 50 and 15. The histogram sliced through the sensitivity data and showed a higher run frequency of 180 between the ranges of 17.2 to 24.3 for the total simulation period of 18 years until 2040. From the histogram, for sensitivity runs, a maximum, minimum, and mean value have been observed to be 96.1, 10, and 33.7, respectively, representing the boundary adequacy of the parameters of the ASD model in extreme conditions. The standard deviation value of 19.5 is well below the IQR, which is 29.8, representing the robustness of the model. The scattered comparative graph of PWFI and plastic waste generation with Sobol sequence pattern shows a lowest and highest value of PWFI of 9.32 and 99.80, respectively, with a highest quantity of a mere 40 million kg plastic waste and a lowest of 15 million kg representing the simulated as well as controlled value range capacity of the built ASD model. The sensitivity graph and confidence interval graph with histogram is shown in Fig. 11, and the comparative scattered graph is shown in Fig. 12.

## Policy simulation results

**Fig. 13:**
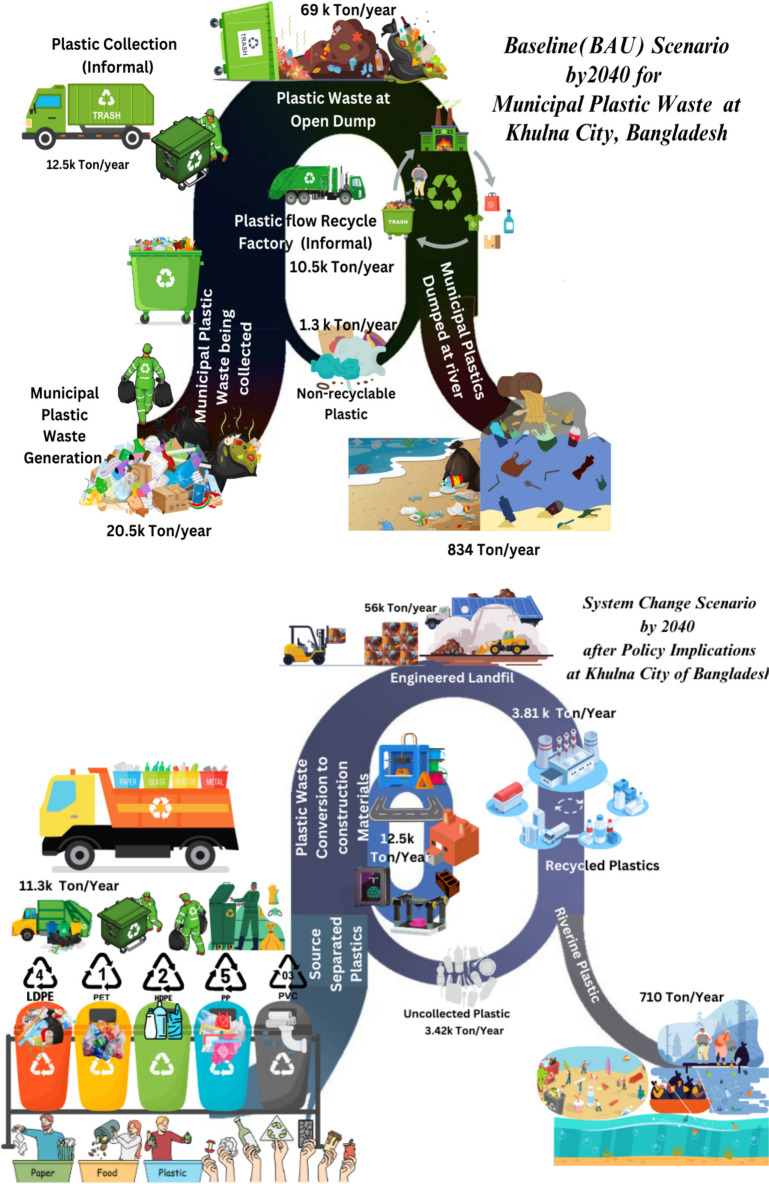
Municipal plastic waste management comparison scenario with baseline and system change (policy implementation)

In order to reduce the municipal plastic waste flow, two consecutive policy simulation results have been analyzed with respect to the baseline (business as usual) scenario. A graphical representation of the improved plastic waste management scenario after changes in the system and operations is shown in Fig. 13. The best-fit policy scenario has been considered in this regard. The aim of this study is briefly stated through the illustration in both quantitative and qualitative form.

### Policy 1 implementation

**Fig. 14:**
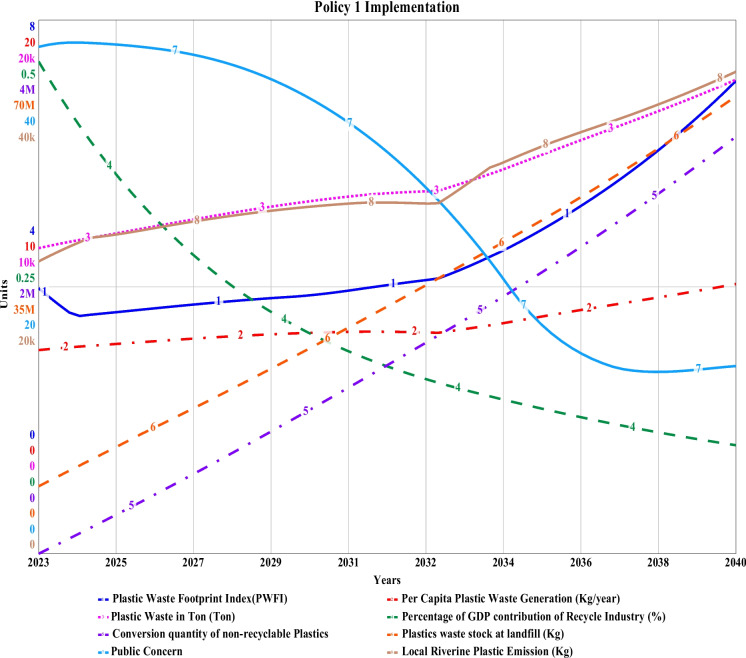
Simulated policy 1 for sustainable plastic waste management of Khulna City

The initial increase in source separation of plastic waste from the point of generation and the increase of source-separated collection, as a simulation result for policy 1, indicates a slight linear drop in the PWFI for 2024 to 3.56 from 3.98. Then, it showed a linear increasing pattern to a consistent value of 3.9 until 2030. But due to an increase in the amount of recyclable and non-recyclable plastic in landfills, secondary disposal sites, near river banks, and in our neighborhood, an abruptly exponential increase has been observed until 2040 to its final value of 7.54 which for the base simulation has been 24. This is because with increased initiatives in plastic waste source separation, reducing waste discharge to rivers and drains, the efficient collection has been successful for the short term, but the plastic waste pile in the landfill and disposal sites has been increasing as a significant portion of this plastic will be single-use non-recyclable plastics which will also be contributing to the increased quantity of riverine discharge. The per capita plastic waste generation however decreased from the base value of 11.6 to 10.3 kg by 2040. On the contrary, an excessive quantity of collected plastic will end up in landfills, secondary disposal areas or nearby water estuaries. Moreover, source separation initiatives will give contentment to the citizens for the short term, and they will be less aware of reducing plastic consumption. As a result, ultimately, the total plastic waste will increase in a stochastic of logarithmic and exponential growth during the simulation period from 11.5 thousand tons in 2023 to 17.8 thousand by 2040, which is almost 3000 tons less if compared to the baseline simulation. Riverine Plastic discharge reduction potential (riverine plastic accumulation) has been the most encouraging factor for this policy measure as it reduced to 36.1 thousand kg by 2040, which is 797 thousand kg less than the baseline plastic waste quantity. However, achieving this goal requires implementing and constructing designated disposal points to the identified 25 nos. hotspot zones, as shown in Fig. 2. Non-recyclable plastic waste conversion and reduction capacity can reach as much as 12.5 million kg by 2040. Though initially the recycling industry’s contribution to regional GDP has been identified to be 0.461%, it kept decreasing to a final value of 0.102%, as there will be a closure of business after a certain time because of government initiatives and recognition. While interviewing the recycle shop owners, we received the impression that it is one of their vital businesses but not the main business and they are trying to relocate these plastic recycling businesses to metal and e-waste recycling for better incentives. Plastic waste conversion is a new concept that can be implemented to convert non-recyclable plastic into valuable construction materials such as 3D printing ingredients, building blocks and bituminous binding materials. After assessing the overall scenario, this study recommends the introduction of plastic conversion technology, not only for Khulna but also for all the big cities in Bangladesh. The conversion initiative considered in this study has the potential to reach a capacity of 3.13 million kg by 2040. Finally, with less plastic waste, public concern regarding this issue will decrease from a value of 38 during 2023 to 14.1 by 2040, which is a real good sign. Though most of these initiatives are successful in the short term, in the long term, they represent less sustainable behavior. Moreover, these policy measures are not adequate enough to cope up with the drastically changing lifestyle of the citizens. Therefore, a second policy has been considered for more efficiency attires. The simulated comparative graph for “Policy 1” is shown in Fig. 14.

### Simulated policy 2 results

**Fig. 15:**
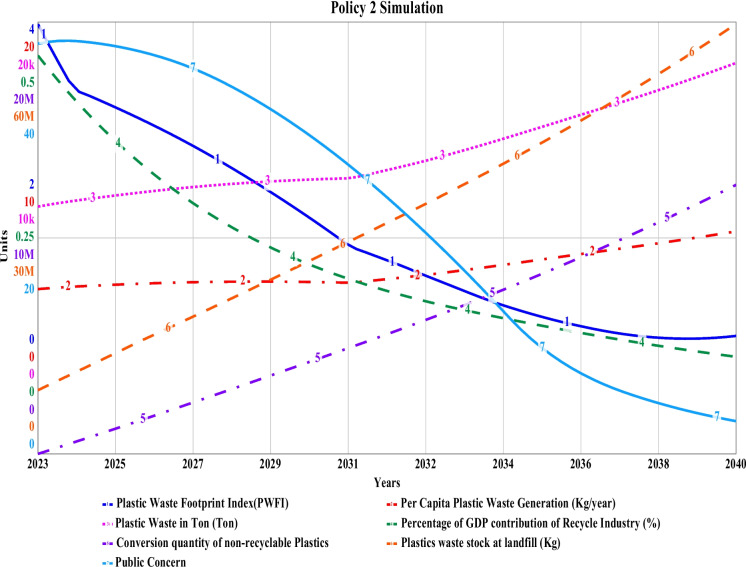
Simulated policy 2 for municipal plastic waste management of Khulna City

Policy 2 simulation, from the learnings about drawbacks of baseline scenario and policy 1, has shown encouraging results. After assigning the measures discussed in Section 3.0, the plastic waste footprint index (PWFI) value reduced exponentially after 2030 to 1.07 by 2040 from an initial value of 3.97 and a linear drop during 2023. This indicates an extensive reduction in the flow quantity of plastic waste around the city. Per capita plastic waste generation remains consistent with an ultimate value of 10.1 kg by 2040 from an initial improved quantity of 7.63 kg. Total plastic waste generation, however, will be slightly reduced to 17.4 thousand tons by 2040, which is 4 thousand tons less than the results obtained from the “policy 1” simulation. Plastic waste pileup at landfills will increase linearly to 56 million kg by 2040 from an initial 8.9 million kg during 2023, which is around 12 million kg less than the baseline scenario. For a knit measure to reduce plastic waste flow around the city, it is imperative to be technologically advanced with the availability of industry-scale plastic processing, sorting, recycling, and conversion technology to utilize and convert the waste into valuable materials such as plastic sand bricks, plastic thin films in road construction. For this policy, the conversion quantity has been found to be 12.5 million kg by 2040, which is three times more than “policy 1.” Though the local riverine plastic discharge graph indicates a more linear increase for the multi-scale graph in Fig. 15, the actual quantity decreases drastically to 7.1 thousand kg by 2040 from the baseline and policy value of 834 thousand kg. The riverine discharge of this coastal city can be improved through extensive awareness campaigns and the construction of waste collection points at hotspot zones, which are being conducted through the scope of some plastic mitigation projects. Most of the large-scale kacha bazars (open fish and vegetable markets) are situated near the banks of the Bhairab and Rupsha rivers in the city, and they are one of the biggest contributors to riverine plastic emissions. To obtain a more reduced quantity of riverine plastic emissions, the market places, shops, and factories must be monitored on a frequent basis. Moreover, while conducting our survey, we found 30 nos. small, medium, and large drain outlets directly connected to the adjacent river. Wiremesh and steel grills are to be set up in those open outlets to prevent the direct deposition of macro plastic into the river. The percentage of the contribution of the local plastic recycling industry will decline exponentially to a final value of 0.112% by 2040, from a starting value of 0.461% due to the closure of informal shops, lack of formal government incentives, tax imposed, and less improved collection methods in the base scenario. Public concern reduced in “S”-shaped pattern to 3.01 by 2040 from an initial concern of 38 during 2023 due to the extensively reduced plastic flow in significant hotspot areas of the city. By observing the stated results, it is quite visible that “policy 2” suits best to ensure the outline for sustainable green growth for this city corporation while ensuring extensive plastic footprint reduction. The representation of results in graphical is shown in Fig. 15.

## Conclusion

In order to reduce the MSPW flow and its footprint, consideration of significant issues ranging from municipal plastic waste generation, collection, mismanagement, riverine discharge, recycling, conversion, energy recovery, financial aspects to public concern, vented reaction, etc., which are independently knitted together has to be prioritized. Therefore, this study aims to evaluate the long-term impact of multiple measures and policies for reducing plastic waste flow and footprints left in city areas, which is the first of its kind, specifically in the context of Bangladesh. The EEIOA concept has been considered for this agent-based system dynamics model for delivering policy measures that can lay the foundation of sustainable policies on a municipal scale. No other study to date has addressed this alarming issue while using the above-stated methodologies and simulation processes. Therefore, this developed ASD model can be used as a simulation laboratory for determining sustainable policy measures for reducing municipal plastic waste flow and managing the ever-increasing plastic waste all over the country. Moreover, countries around the world have imposed strict plastic pollution control policies, whereas developing countries such as Bangladesh are facing difficulties in the absolute implementation of the regulations imposed. In contrast to the previous research on the plastic waste footprint that mainly focused on the per capita waste generation measurement method, the development of the ASD model considered significant macro as well as micro factors such as source separation of plastic, collection of source-separated plastics, plastic conversion, economic aspect, and riverine plastic flow reduction through a single platform, which is a novel approach for not only municipalities of Bangladesh but also in south-east Asia. However, this study has limitations regarding the unavailability of datasets on the detailed economic analysis from a circularity perspective, such as the budget allocation for plastic waste management, wages and related transportation cost of plastic wastes to ASD, recycle shops and landfills, the livelihood of the workers in the sector, etc. Apart from the annual turnover of recycle shops, very few economic datasets have been available due to the informal practice of plastic management in the city. Moreover, the level of dynamic complexities because of non-linearity and specific delays of factors are inherent aspects that may create challenges for decision-makers. Furthermore, the GHG emission due to incineration and reduction due to the recycling process has not been considered due to the lack of available on-site assessment with unit values for this spatial aspect. Furthermore, considering microplastic and nano-plastic materials in this PWFI will result in a deviated concentration of microplastic flow reduction. As micro and nano-plastic originate from macro plastic materials, it has been proposed that a further detailed study on the degradation behavior of macro plastic (physically and chemically) is to be assessed, and a different indexing method specifically concentrated on micro and nano-plastic has to be quantified. The difference can be exampled generically similar to Ultra violet index and Light pollution index (Fang et al., 2023). It is because both have invisible and visible irradiance spectra but are different in terms of definition and injurious effects on humans as well as biodiversity (Solano Lamphar & Kocifaj, 2013). However, this study can be the baseline for conducting further studies on GHG as well as energy reduction potentials from a regional and global perspective. In-spite of these limitations, the ASD model represents a more detailed policy scenario for sustainable plastic waste management of Khulna City in the long run. Finally, after the impact of the proposed policies has been tested, the results indicate “policy 2” to be adequate enough to achieve sustainable municipal plastic management tires in the long run. It is to be noted that plastic conversion technology with environmental and economic feasibility is to be conducted before setting up such facilities, specifically near the river bank industrial zones. It is proposed while keeping in mind the potential microplastic pollution scenario that may emerge from such establishment. This will be conducted in a further improvised study concentrated on quantum aspects like microplastics, its economic, ecologic and health impact. Despite these boundaries, the proposed ASD model and its quantitative approach will play a vital role for the decision makers in choosing the most sustainable MPSW management strategies for green development. Finally, the developed ASD model can be used for further studies on determining microplastic footprint and alleviation methods to ensure a healthy, green and sustainable plastic free environment alongside efficient economic circularity.

## Supplementary Information

Below is the link to the electronic supplementary material.

Supplementary file1 (DOCX 6889 KB)

## Data Availability

All data generated or analyzed during this study are available upon request. Some of the major datasets are available within the article and in the supplementary materials.
