# Correction to: Forecasting municipal solid plastic waste generation and management policy using system dynamics: a case study of Khulna City in Bangladesh

**DOI:** 10.1007/s10661-024-12907-5

**Published:** 2024-08-01

**Authors:** Islam M. Rafizul, Eckhard Kraft, Thomas Haupt, S. M. Rafew

**Affiliations:** 1 Department of Civil Engineering, Khulna University of Engineering and Technology, Khulna, 9203 Bangladesh; 2 Biotechnology in Resources Management, Faculty of Civil Engineering, Bauhaus-Universität Weimar, Coudraystr. 7, 99423 Weimar, Germany

**Correction to: Environ Monit Assess (2024) 196:544**

10.1007/s10661-024-12684-1

The article Forecasting municipal solid plastic waste generation and management policy using system dynamics: a case study of Khulna City in Bangladesh, written by Islam M. Rafizul, Eckhard Kraft, Thomas Haupt and S. M. Rafew, was originally published Online First without Open Access. After publication in volume 196, issue 6, article ID 544 the author decided to opt for Open Choice and to make the article an Open Access publication. Therefore, the copyright of the article has been changed to © The Author(s) 2024 and the article is forthwith distributed under the terms of the Creative Commons Attribution 4.0 International License, which permits use, sharing, adaptation, distribution and reproduction in any medium or format, as long as you give appropriate credit to the original author(s) and the source, provide a link to the Creative Commons licence, and indicate if changes were made. The images or other third party material in this article are included in the article’s Creative Commons licence, unless indicated otherwise in a credit line to the material. If material is not included in the article’s Creative Commons licence and your intended use is not permitted by statutory regulation or exceeds the permitted use, you will need to obtain permission directly from the copyright holder. To view a copy of this licence, visit http://creativecommons.org/licenses/by/4.0/.

The original article has been corrected.

