# Supplementary material for: Forecasting municipal solid plastic waste generation and management policy using system dynamics: a case study of Khulna City in Bangladesh
Source: Environ Monit Assess. 2024 May 14;196(6):544. doi: 10.1007/s10661-024-12684-1 (PMC11530515; doi:10.1007/s10661-024-12684-1)

**Forecasting Municipal Solid Plastic Waste Generation and Management Policy using System Dynamics: A Case Study of Khulna City in Bangladesh**

Islam M Rafizul^1^, Eckhard Kraft^2^, Thomas Haupt^2^, ^*^S. M. Rafew^1^

^1^Professor, Department of Civil Engineering, Khulna University of Engineering and

Technology, Khulna-9203, Bangladesh.

^2^ Professor, Biotechnology in Resources Management, Faculty of Civil Engineering, Bauhaus-Universität Weimar, Coudraystr. 7, Weimar 99423, Germany.

^2^Professor, Biotechnology in Resources Management, Faculty of Civil Engineering,

Bauhaus-Universität Weimar, Coudraystr. 7, Weimar 99423, Germany.

^1^M.Sc. in Civil Engineering, Department of Civil Engineering, Khulna University of

Engineering and Technology, Khulna-9203, Bangladesh.

*Corresponding author’s email: [rafew1801552@stud.kuet.ac.bd](mailto:rafew1801552@stud.kuet.ac.bd)

(ORCID: [0000-0003-2724-6089](https://orcid.org/0000-0003-2724-6089))

**Supplementary File**

**Appendix A**

**Baseline Code for the System Dynamics Model**

Top-Level Model:

"Conversion_quantity_of_non-recyclable_Plastics"(t) = "Conversion_quantity_of_non-recyclable_Plastics"(t - dt) + (Inflow_of_Plastic_for_conversion) * dt

INIT "Conversion_quantity_of_non-recyclable_Plastics" = 0

INFLOWS:

Inflow_of_Plastic_for_conversion = "Non-Recyclable_Plastics"*0.0 {UNIFLOW}

GDP(t) = GDP(t - dt) + (GDP_Growth) * dt

INIT GDP = 155193400000

UNITS: BDT

INFLOWS:

GDP_Growth = GDP*(GDP_Growth_Rate/100) {UNIFLOW}

UNITS: BDT/Years

Number_of_Recycle_Shops(t) = Number_of_Recycle_Shops(t - dt) + (Increase_rate_of_Recycle_Shops - Closed_Recycle_shops) * dt

INIT Number_of_Recycle_Shops = 35

INFLOWS:

Increase_rate_of_Recycle_Shops = Increment_rate_of_Recycle_Shops/"Time_req._for_New_Recycle_shop"*0 {UNIFLOW}

OUTFLOWS:

Closed_Recycle_shops = (Number_of_Recycle_Shops/Time_for_closing)*0.5 {UNIFLOW}

Plastics_waste_stock_at_landfill(t) = Plastics_waste_stock_at_landfill(t - dt) + (Plastic_waste_deposition_rate_at_landfill - Incineration_and_Informal_Collection_at_Landfill - Plastic_waste_used_for_WTE) * dt {NON-NEGATIVE}

INIT Plastics_waste_stock_at_landfill = 8853066.6216

UNITS: Kg

INFLOWS:

Plastic_waste_deposition_rate_at_landfill = Plastic_Waste_transported_at_Landfill {UNIFLOW}

UNITS: kg/year

OUTFLOWS:

Incineration_and_Informal_Collection_at_Landfill = Plastics_waste_stock_at_landfill*0.002 {UNIFLOW}

UNITS: kg/year

Plastic_waste_used_for_WTE = DELAY(Plastic_required_for_Installed_Pyrolysis_Plant, Delay_Time, 1.5) {UNIFLOW}

UNITS: kg/year

Population(t) = Population(t - dt) + (Population_Growth) * dt {NON-NEGATIVE}

INIT Population = 1500689

UNITS: People

INFLOWS:

Population_Growth = Population*Population_Growth_Rate {UNIFLOW}

UNITS: People/Years

Public_Concern(t) = Public_Concern(t - dt) + (Concern_rate - Vented_Reaction) * dt {NON-NEGATIVE}

INIT Public_Concern = 38

INFLOWS:

Concern_rate = PWFI_vs_Concern_Rate {UNIFLOW}

OUTFLOWS:

Vented_Reaction = Public_Concern/Dissipation_Time {UNIFLOW}

Recyclable_Plastic_Stock(t) = Recyclable_Plastic_Stock(t - dt) + (Inflow_of_Plastic_in_Recycle_Shops - Outflow_for_Export_of_Recycled_materials) * dt {NON-NEGATIVE}

INIT Recyclable_Plastic_Stock = 2686982.57171

UNITS: Kg

INFLOWS:

Inflow_of_Plastic_in_Recycle_Shops = Recyclable_Portion_of_collected_Plastic {UNIFLOW}

UNITS: kg/year

OUTFLOWS:

Outflow_for_Export_of_Recycled_materials = Total_Recycling_Capacity {UNIFLOW}

UNITS: kg/year

Riverine_Plastic_Discharge_Accumulation(t) = Riverine_Plastic_Discharge_Accumulation(t - dt) + (Local_riverine_plastic_emission_rate - Plastic_Flowing_Downstream) * dt

INIT Riverine_Plastic_Discharge_Accumulation = 1297200*0.70

UNITS: Kg

INFLOWS:

Local_riverine_plastic_emission_rate = Local_Riverine_Plastic_Emission+(Local_Riverine_Plastic_Emission/Riverine_Plastic_Discharge_Accumulation) {UNIFLOW}

UNITS: kg/year

OUTFLOWS:

Plastic_Flowing_Downstream = Riverine_Plastic_Discharge_Accumulation*0.52 {UNIFLOW}

UNITS: kg/year

"\"Avg._Recycling_Capacity\"_(Base_value)" = Base_Recycling_Capacity/Base_Number_of_Recycle_Shops

UNITS: kg/shop

"Time_req._for_New_Recycle_shop" = 10

"Weighted_Avg._1" = 0.5

"Weighted_Avg._2" = 0.5

"Weighted_Avg._3" = 0.5

"Weighted_Avg._4" = 0.05

"Weighted_Avg._5" = 0.5

"Weighted_Avg._6" = 0.5

Annual_Turnover_of_Recycle_Shops = Average_annual_turnover_per_recycle_shop*Number_of_Recycle_Shops

Average_annual_turnover_per_recycle_shop = "Baseline_Annual_Turn-over_of_the_Recycle_Shops"/Base_Number_of_Recycle_Shops

UNITS: BDT/shop

Base_Number_of_Recycle_Shops = 35

Base_Recycling_Capacity = (830.0*1000)*12

UNITS: kg

Base_Value_for_Population = 1500689

Base_Value_for_riverine_plastic_emission = 635637.848332

UNITS: Kg

"Base_Value_of_Non-recyclable_Plastic_waste" = 123457.519345

UNITS: Kg

Base_Value_of_Plastic_waste_conversion = 1118979.21171

UNITS: Kg

Base_Value_of_Plastic_Waste_Stock_at_Landfill = 8853066.6216

UNITS: Kg

Base_Value_of_Plastic_waste_with_no_collection = 2946780.13343

UNITS: kg

Base_Value_of_Recyclable_Plastic_waste = 2686982.57171

UNITS: Kg

"Baseline_Annual_Turn-over_of_the_Recycle_Shops" = 715806016.8

UNITS: BDT

Delay_Time = 2.5

UNITS: Years

Dissipation_Time = GRAPH(TIME)

(2023.00, 11.10), (2025.83333333, 9.24150814259), (2028.66666667, 6.74474848608), (2031.50, 4.43603509826), (2034.33333333, 2.25070212201), (2037.16666667, 2.00213188025), (2040.00, 1.3296815)

Fraction_of_Plastic_Recycled_from_the_Recyclable_portion = (Base_Recycling_Capacity/Recyclable_Portion_of_collected_Plastic)*100

UNITS: %

GDP_Growth_Rate = GRAPH(TIME)

(1.000, 6.10), (2.400, 6.30), (3.800, 7.25), (5.200, 7.15), (6.600, 7.00), (8.000, 8.00)

"Incinerated_amount_of_the_Non-recyclable_waste" = "Non-Recyclable_Portion_of_the_Collected_Plastic"*0.01

Income_Multiplier = GRAPH(Per_Capita_GDP)

(100000, 113.15), (180000, 131.087142857), (260000, 147.51952381), (340000, 163.205178571), (420000, 178.666283367), (500000, 194.198296421), (580000, 209.886029972), (660000, 225.626652791), (740000, 241.159623221), (820000, 256.103552734), (900000, 270.0)

Increment_rate_of_Recycle_Shops = GRAPH(TIME)

(2023.00, 0.00), (2026.40, 5.6935961243), (2029.80, 13.3625090102), (2033.20, 20.3061006499), (2036.60, 21.7794705058), (2040.00, 25.00)

UNITS: nos.

"Informal_Collection_of_non-separated_waste" = DELAY(("Non-separated_Plastic_waste"*0.10), 1.2)

Informal_collection_of_source_separated_plastic_waste = Source_Separated_Plastic_waste*0.78

UNITS: kg/year

Local_Riverine_Plastic_Emission = Plastic_waste_with_no_collection_facility*0.2157058957744450

UNITS: Kg

MSW_Generation_in_Tons_per_day = (Total_MSW_Generation/1000)/365

UNITS: Tons

"Non-Recyclable_Plastics" = "Non-Recyclable_Portion_of_the_recyclable_collection"+"Non-Recyclable_Portion_of_the_Collected_Plastic"

UNITS: kg

"Non-Recyclable_Portion_of_the_Collected_Plastic" = (Informal_collection_of_source_separated_plastic_waste+"Informal_Collection_of_non-separated_waste")*0.0953169

UNITS: Kg

"Non-Recyclable_Portion_of_the_recyclable_collection" = Recyclable_Portion_of_collected_Plastic*0.0081927710843373

UNITS: kg

"Non-separated_Plastic_waste" = IF((Plastic_Waste_Generation-Source_Separated_Plastic_waste)<Plastic_Waste_Generation)THEN(Plastic_Waste_Generation-Source_Separated_Plastic_waste)ELSE(0)

UNITS: Kg

Per_Capita_GDP = GDP/Population

UNITS: BDT/People

Per_capita_MSW_Generation = Income_Multiplier*Per_Capita_MSW_normalized

UNITS: Kg/People/Year

Per_Capita_MSW_normalized = 1.5

Per_Capita_Plastic_Waste_Generation = (Plastic_Waste_Generation/Population)

UNITS: Kg/year

Percentage_of_GDP_contribution_of_Recycle_Industry = (Annual_Turnover_of_Recycle_Shops/GDP)*100

UNITS: %

Plastic_required_for_Installed_Pyrolysis_Plant = 182500

UNITS: kg

"Plastic_Waste_Footprint_Index(PWFI)" = (("Weighted_Avg._1"*(Recyclable_Plastic_Stock/Base_Value_of_Recyclable_Plastic_waste))+("Weighted_Avg._2"*("Non-Recyclable_Plastics"/"Base_Value_of_Non-recyclable_Plastic_waste"))+("Weighted_Avg._3"*(Plastic_waste_with_no_collection_facility/Base_Value_of_Plastic_waste_with_no_collection))+("Weighted_Avg._4"*(Plastics_waste_stock_at_landfill/Base_Value_of_Plastic_Waste_Stock_at_Landfill))+"Weighted_Avg._5"*(Local_Riverine_Plastic_Emission/Base_Value_for_riverine_plastic_emission)-("Weighted_Avg._6"*("Conversion_quantity_of_non-recyclable_Plastics"/Base_Value_of_Plastic_waste_conversion)))*Population_Ratio

Plastic_Waste_Generation = Total_MSW_Generation*(Plastic_waste_generation_rate/100)*Plastic_waste_Generation_Normalized

UNITS: Kg/year

Plastic_waste_Generation_Normalized = 0.808

Plastic_waste_generation_rate = GRAPH(Public_Pressure_Multiplier)

(1.000, 7.125), (2.000, 6.91963543523), (3.000, 6.2062789913), (4.000, 5.81723374807), (5.000, 5.89615710417)

Plastic_Waste_in_Ton = Plastic_Waste_Generation/1000

UNITS: Ton

Plastic_Waste_transported_at_Landfill = DELAY(("Non-Recyclable_Portion_of_the_Collected_Plastic"+(Uncollected_Plastic_Waste_from_source*.50)), 1.3)

Plastic_waste_with_no_collection_facility = Plastic_Waste_Generation-(Plastic_Waste_transported_at_Landfill+Recyclable_Portion_of_collected_Plastic+"Incinerated_amount_of_the_Non-recyclable_waste")

UNITS: kg

Population_Growth_Rate = (0.93/100)

Population_Ratio = Population/Base_Value_for_Population

Public_Pressure_Multiplier = GRAPH(Public_Concern)

(1.0, 0.000), (20.9, 1.67915455835), (40.8, 2.80472551921), (60.7, 3.5592182975), (80.6, 4.06496993138), (100.5, 4.40398538989), (120.4, 4.63123424764), (140.3, 4.78356371243), (160.2, 4.88567320628), (180.1, 4.9541192469), (200.0, 5.000)

PWFI_vs_Concern_Rate = GRAPH("Plastic_Waste_Footprint_Index(PWFI)")

(0.00, 1.00392763864), (2.72727272727, 2.46733960401), (5.45454545455, 5.97835484188), (8.18181818182, 14.0110507075), (10.9090909091, 30.5474900253), (13.6363636364, 58.2420882889), (16.3636363636, 91.7579117111), (19.0909090909, 119.452509975), (21.8181818182, 135.988949292), (24.5454545455, 144.021645158), (27.2727272727, 147.532660396), (30.00, 148.996072361)

Recyclable_Portion_of_collected_Plastic = (Informal_collection_of_source_separated_plastic_waste+"Informal_Collection_of_non-separated_waste")-"Non-Recyclable_Portion_of_the_Collected_Plastic"

UNITS: Kg

Riverine_emission_in_Ton = Local_Riverine_Plastic_Emission/1000

Source_Separated_in_Ton = Source_Separated_Plastic_waste/1000

Source_Separated_Plastic_waste = Plastic_Waste_Generation*(Source_Separation)

Source_separated_plastics_in_Ton = Informal_collection_of_source_separated_plastic_waste/1000

Source_Separation = GRAPH((TIME)*Public_Pressure_Multiplier)

(2.65, 0.3300), (5.66, 0.381338911733), (8.91, 0.424372413757), (12.48, 0.461554288436), (16.45, 0.494874064133), (20.94, 0.525883583607), (21.96, 0.555723572397), (31.04, 0.585150207218), (37.17, 0.614561684351), (43.50, 0.644024788031), (50.16, 0.673301458839), (56.64, 0.701875362093), (62.79, 0.728978456239), (69.02, 0.753617561242), (75.00, 0.774600926973), (93.915625, 0.790564801605), (100.00, 0.8000)

Time_for_closing = 8

Total_MSW_Generation = Population*Per_capita_MSW_Generation

UNITS: Kg/Year

Total_Recycling_Capacity = Number_of_Recycle_Shops*"\"Avg._Recycling_Capacity\"_(Base_value)"

Uncollected_Plastic_Waste_from_source = Plastic_Waste_Generation-(Informal_collection_of_source_separated_plastic_waste+"Informal_Collection_of_non-separated_waste")

UNITS: Kg

Waste_other_than_Plastics = Total_MSW_Generation-Plastic_Waste_Generation

UNITS: Kg/Year

{ The model has 85 (85) variables (array expansion in parens).

In root model and 0 additional modules with 6 sectors.

Stocks: 8 (8) Flows: 14 (14) Converters: 63 (63)

Constants: 25 (25) Equations: 52 (52) Graphicals: 8 (8)

}

**Appendix B**

**Table: Annual Turnover for Recycle shops in Khulna city**

| **SL.** | **Shop Identification (Zone + Number)** | **Name of the Shops** | ***Monthly Turnover (BDT)*** | ***Annual Turnover (BDT)*** |
| --- | --- | --- | --- | --- |
| 1 | Lobonchora Shop-1 | Khulna Plastic | 50000 | 600000 |
| 2 | Lobonchora Shop-2 | Saroar Recycling Factory | 1700000 | 20400000 |
| 3 | Lobonchora Shop-3 | M/S Jabbar Enterprise - Abdullah Plastic | 120000 | 1440000 |
| 4 | Lobonchora Shop-4 | Islam Plastic Products | 500000 | 6000000 |
| 5 | Khalishpur Shop-1 | Ma Babar Doya Plastic | 400000 | 4800000 |
| 6 | Khalishpur Shop-2 | Jahid Enterprise | 700000 | 8400000 |
| 7 | Khalishpur Shop-3 | Jihad Plastic Cutting Center | 2000000 | 24000000 |
| 8 | Khalishpur Shop-4 | Anika Plastics | 4000000 | 48000000 |
| 9 | Khalishpur Shop-5 | Messrs Shorif and Brothers | 7000000 | 84000000 |
| 10 | Khalishpur Shop-6 | Messrs Sumaiya Recycling Pet Flakes | 3000000 | 36000000 |
| 11 | Khalishpur Shop-7 | Messrs Polymer Cut and Shape Company | 400000 | 4800000 |
| 12 | Khalishpur Shop-8 | Al Madina Traders | 2000000 | 24000000 |
| 13 | Khalishpur Shop-9 | Asma Akter Lipi Enterprise | 200000 | 2400000 |
| 14 | Khalishpur Shop-10 | Tithi Sneha PET Flakes | 1500000 | 18000000 |
| 15 | Zero Point Shop-1 | Mayer Dowa Metal and Hasib Traders | 900000 | 10800000 |
| 16 | Zero Point Shop-2 | Aladin Plastic Center | ------ | ------ |
| 17 | Zero Point Shop-3 | S R Poultry Plastic Equipments | 1500000 | 18000000 |
| 18 | Zero Point Shop-4 | J K Trade International | 5000000 | 60000000 |
| 19 | Zero Point Shop-5 | Mayer Dowa Plastic Recycling Center | 150000 | 450000 |
| 20 | Zero Point Shop-6 | Wasifa Plastic Cutting Center | 500000 | 6000000 |
| 21 | Sonadanga Shop-1 | Bismillah Plastic | 6500000 | 78000000 |
| 22 | Sonadanga Shop-2 | Madina Plastic | 600000 | 7200000 |
| 23 | Sonadanga Shop-3 | Messrs Diya Enterprise | 800000 | 9600000 |
| 24 | Sonadanga Shop-4 | Mayer Dowa Enterprise | 1500000 | 18000000 |
| 25 | Sonadanga Shop-5 | Messrs Rahman Enterprise | 700000 | 8400000 |
| 26 | Sonadanga Shop-6 | Messrs J.N. PET Flakes | 1000000 | 12000000 |
|  | Total | | | 511290012 |

**Appendix C**

**1. List of Supplementary Table and Figures**

Supplementary Table 1: Waste Generation rate of different socio-economic locations of Khulna city

| **Socio-economic condition** | **Waste generation Rate (kg/cap/Day)** | | | | | | | | |
| --- | --- | --- | --- | --- | --- | --- | --- | --- | --- |
|  | **Ward 1** | **Ward 2** | **Ward 5** | **Ward 9** | **Ward 11** | **Ward 16** | **Ward 17** | **Ward 21** | **Ward 24** |
| **Low (<10000)** | 0.27 | 0.30 | 0.29 | 0.34 | 0.33 | 0.33 | 0.32 | 0.31 | 0.32 |
| **Lower Middle** (10000-20000) | 0.33 | 0.35 | 0.36 | 0.39 | 0.40 | 0.44 | 0.38 | 0.39 | 0.38 |
| **Middle** (20000-30000) | 0.39 | 0.43 | 0.41 | 0.55 | 0.53 | 0.52 | 0.45 | 0.49 | 0.45 |
| **Higher Middle** (3000-40000) | 0.49 | 0.52 | 0.50 | 0.67 | 0.63 | 0.68 | 0.57 | 0.55 | 0.51 |
| **Higher** (>40000) | 0.56 | 0.64 | 0.55 | 0.74 | 0.71 | 0.76 | 0.62 | 0.69 | 0.60 |
| **Average** | 0.41 | 0.45 | 0.42 | 0.54 | 0.52 | 0.55 | 0.47 | 0.49 | 0.45 |


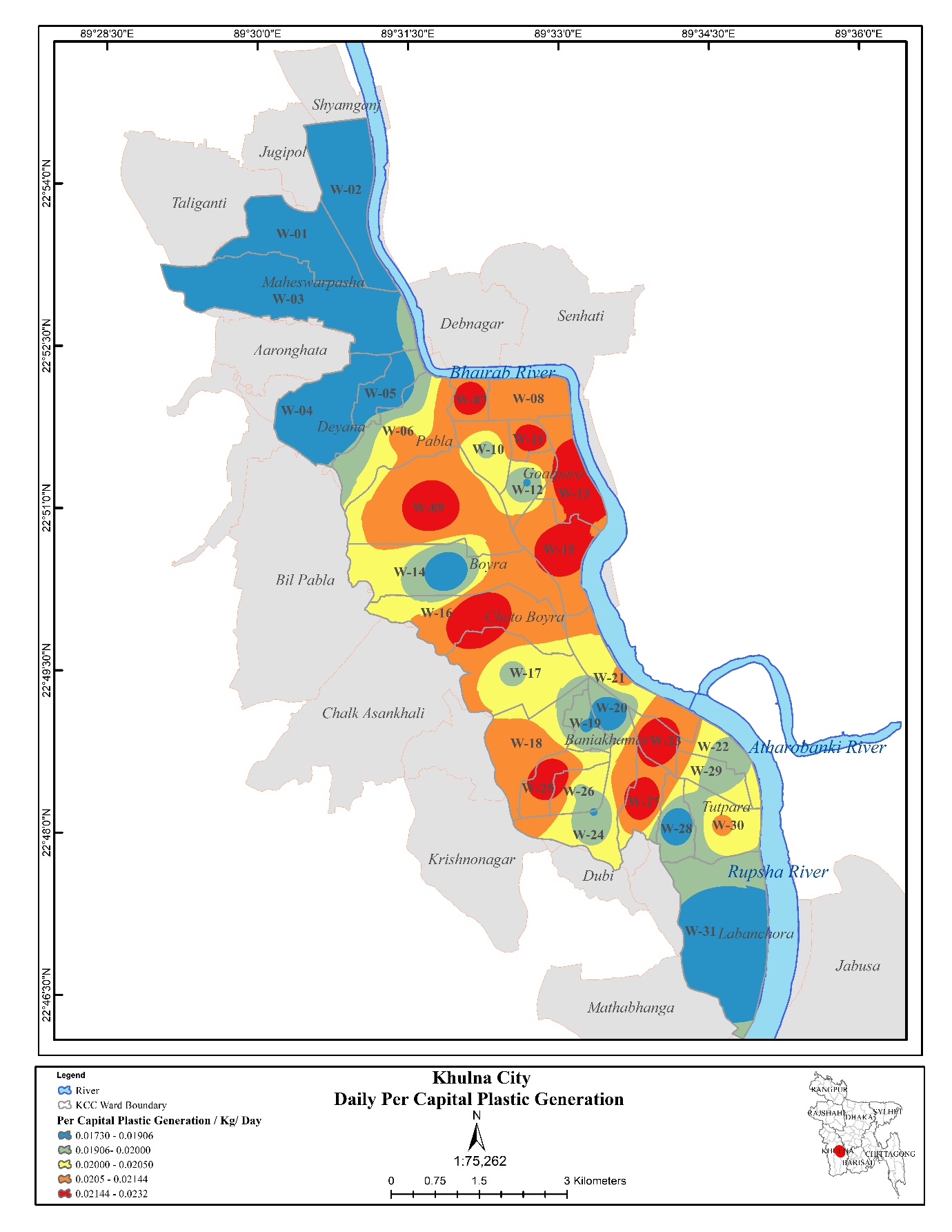


Supplementary Fig. 1: Municipal ward wise distribution of per capita plastic waste generation

*baseline scenario for Khulna city*


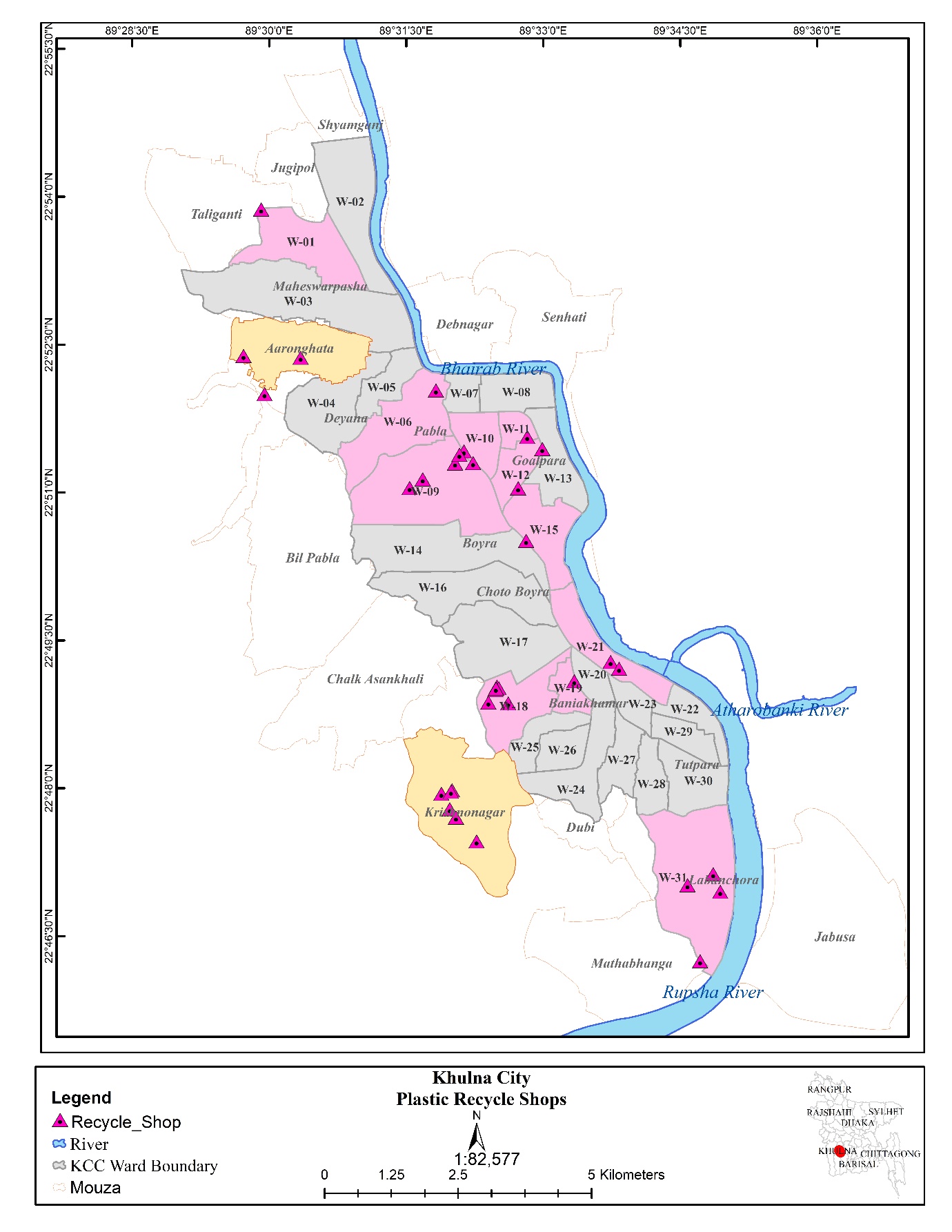


Supplementary Fig. 2: Plastic Recycle Shop location of Khulna City

Supplementary Fig. 3: A sample of ongoing baseline simulation at time step of 2038.25

**2. Sample Photos during Data Collection & Experiment**

**
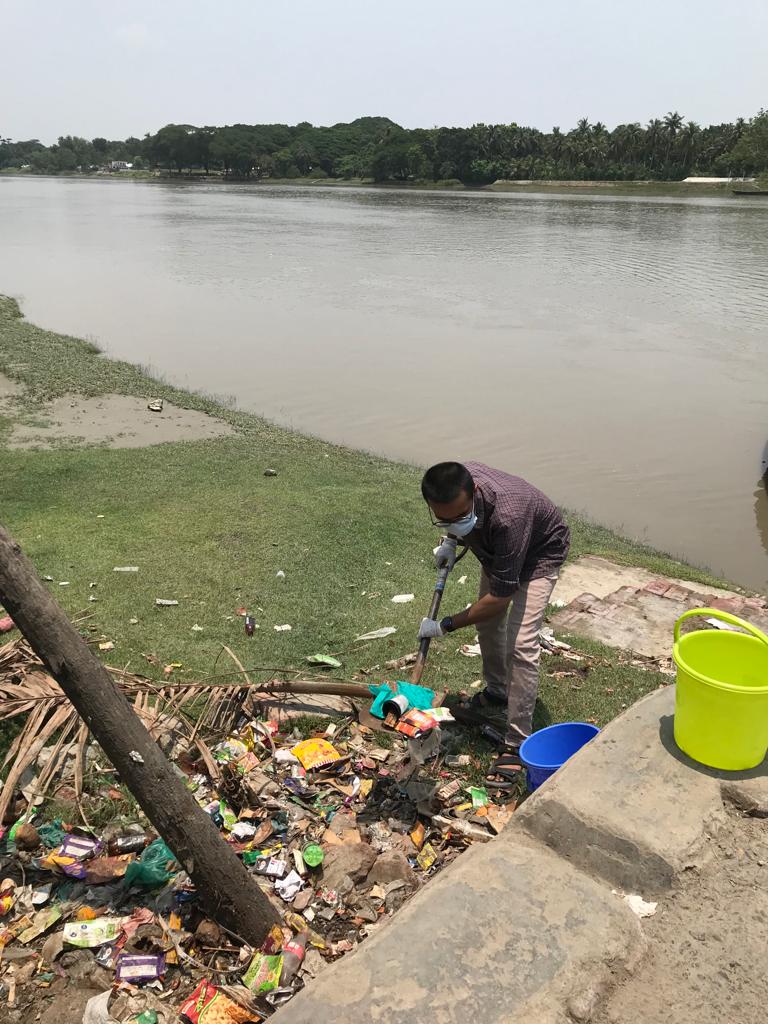

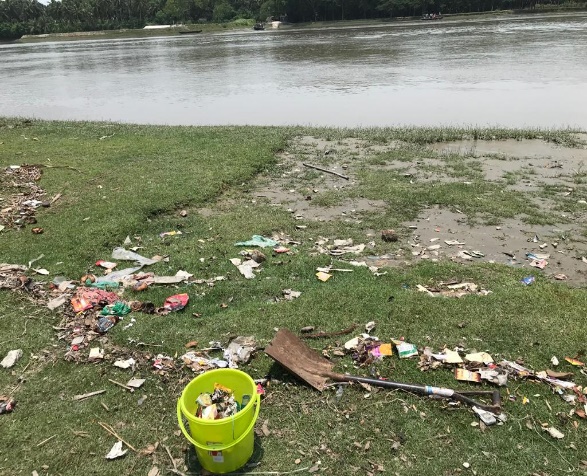

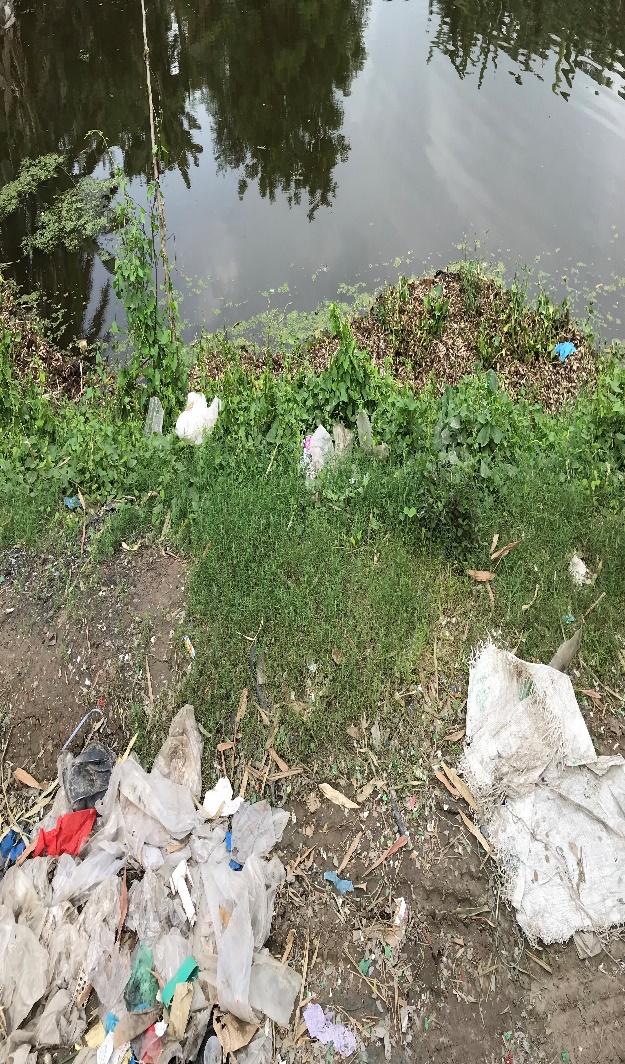
**


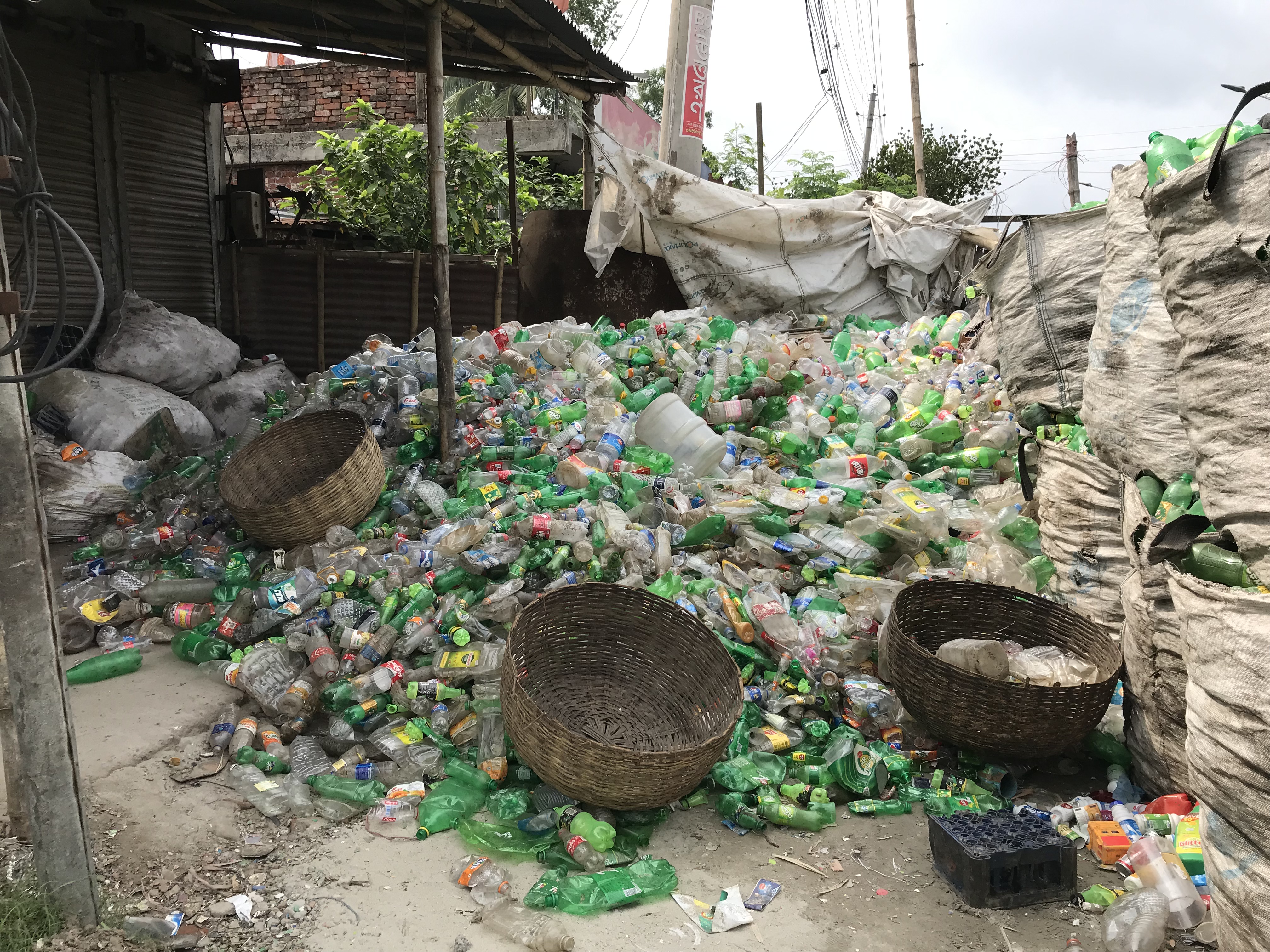

Supplement: Supplementary file 1 — Supplementary file1 (DOCX 6889 KB) [file 10661_2024_12684_MOESM1_ESM.docx]
